# Single-cell transcriptomics reveals regulators underlying immune cell diversity and immune subtypes associated with prognosis in nasopharyngeal carcinoma

**DOI:** 10.1038/s41422-020-0374-x

**Published:** 2020-07-20

**Authors:** Yu-Pei Chen, Jian-Hua Yin, Wen-Fei Li, Han-Jie Li, Dong-Ping Chen, Cui-Juan Zhang, Jia-Wei Lv, Ya-Qin Wang, Xiao-Min Li, Jun-Yan Li, Pan-Pan Zhang, Ying-Qin Li, Qing-Mei He, Xiao-Jing Yang, Yuan Lei, Ling-Long Tang, Guan-Qun Zhou, Yan-Ping Mao, Chen Wei, Ke-Xu Xiong, Hong-Bo Zhang, Shi-Da Zhu, Yong Hou, Ying Sun, Michael Dean, Ido Amit, Kui Wu, Dong-Ming Kuang, Gui-Bo Li, Na Liu, Jun Ma

**Affiliations:** 1 Department of Radiation Oncology, State Key Laboratory of Oncology in South China, Collaborative Innovation Center for Cancer Medicine, Guangdong Key Laboratory of Nasopharyngeal Carcinoma Diagnosis and Therapy, Sun Yat-sen University Cancer Center, Guangzhou, Guangdong 510060 China; 2 BGI-Shenzhen, Shenzhen, Guangdong 518120 China; 3 CAS Key Laboratory of Quantitative Engineering Biology, Shenzhen Institute of Synthetic Biology, Shenzhen Institutes of Advanced Technology, Chinese Academy of Sciences, Shenzhen, Guangdong 518055 China; 4 MOE Key Laboratory of Gene Function and Regulation, School of Life Sciences, Sun Yat-sen University, Guangzhou, Guangdong 510275 China; 5 Key Laboratory for Stem Cells and Tissue Engineering, Ministry of Education and The Department of Histology and Embryology of Zhongshan School of Medicine, Sun Yat-sen University, Guangzhou, Guangdong 510060 China; 6 Laboratory of Translational Genomics, Division of Cancer Epidemiology and Genetics, National Cancer Institute, Gaithersburg, MD USA; 7 Department of Immunology, Weizmann Institute of Science, Rehovot, Israel; 8 Shenzhen Key Laboratory of Single-Cell Omics, BGI-Shenzhen, Shenzhen, Guangdong 518120 China

**Keywords:** Cancer microenvironment, Head and neck cancer

## Abstract

Nasopharyngeal carcinoma (NPC) is an aggressive malignancy with extremely skewed ethnic and geographic distributions. Increasing evidence indicates that targeting the tumor microenvironment (TME) represents a promising therapeutic approach in NPC, highlighting an urgent need to deepen the understanding of the complex NPC TME. Here, we generated single-cell transcriptome profiles for 7581 malignant cells and 40,285 immune cells from fifteen primary NPC tumors and one normal sample. We revealed malignant signatures capturing intratumoral transcriptional heterogeneity and predicting aggressiveness of malignant cells. Diverse immune cell subtypes were identified, including novel subtypes such as *CLEC9A*^+^ dendritic cells (DCs). We further revealed transcriptional regulators underlying immune cell diversity, and cell–cell interaction analyses highlighted promising immunotherapeutic targets in NPC. Moreover, we established the immune subtype-specific signatures, and demonstrated that the signatures of macrophages, plasmacytoid dendritic cells (pDCs), *CLEC9A*^+^ DCs, natural killer (NK) cells, and plasma cells were significantly associated with improved survival outcomes in NPC. Taken together, our findings represent a unique resource providing in-depth insights into the cellular heterogeneity of NPC TME and highlight potential biomarkers for anticancer treatment and risk stratification, laying a new foundation for precision therapies in NPC.

## Introduction

Nasopharyngeal carcinoma (NPC) is a unique subtype of head and neck cancers with an extremely unbalanced endemic distribution; it is highly prevalent in East and Southeast Asia, especially southern China, and North and East Africa.^1^ Etiologically, NPC is highly correlated with Epstein–Barr virus (EBV) infection and is pathologically characterized by heavy infiltration of immune cells around and within tumor lesions,^2,3^ suggesting the existence of a remarkably complex tumor microenvironment (TME) in NPC.

These specific features of the TME indicate the potential benefits of immunotherapy in NPC. Indeed, the inhibition of the immune checkpoint axis programmed cell death protein 1 (PD-1)/PD-ligand 1 (PD-L1) has achieved clinical benefit in NPC patients; nevertheless, the response rate to anti-PD-1 therapies in NPC patients is only 20%–30%.^4–6^ This highlights an unmet clinical need to deepen the understanding of the TME in NPC to identify therapeutic targets and reliable biomarkers for risk stratification. However, current strategies for genomic/transcriptomic analyses of NPC are primarily based on bulk samples from these often small-sized tumors,^7–10^ and these approaches therefore lack the resolution and accuracy to depict the complex heterogeneity of the TME.

Recent advances in single-cell RNA sequencing (scRNA-seq) are opening a new way for evaluating transcriptional features with cellular resolution in various types of cancers.^11–14^ This study represents a comprehensive and unique resource revealing the cellular heterogeneity of NPC TME at single-cell resolution. We uncovered malignant signatures capturing intratumoral heterogeneity. We also identified transcriptional regulators underlying immune cell diversity and established immune subtype-specific signatures associated with prognosis, thereby providing in-depth insights into the multicellular ecosystem of NPC.

## Results

### Single-cell expression atlas and cell typing of the TME in NPC

**Fig. 1:**
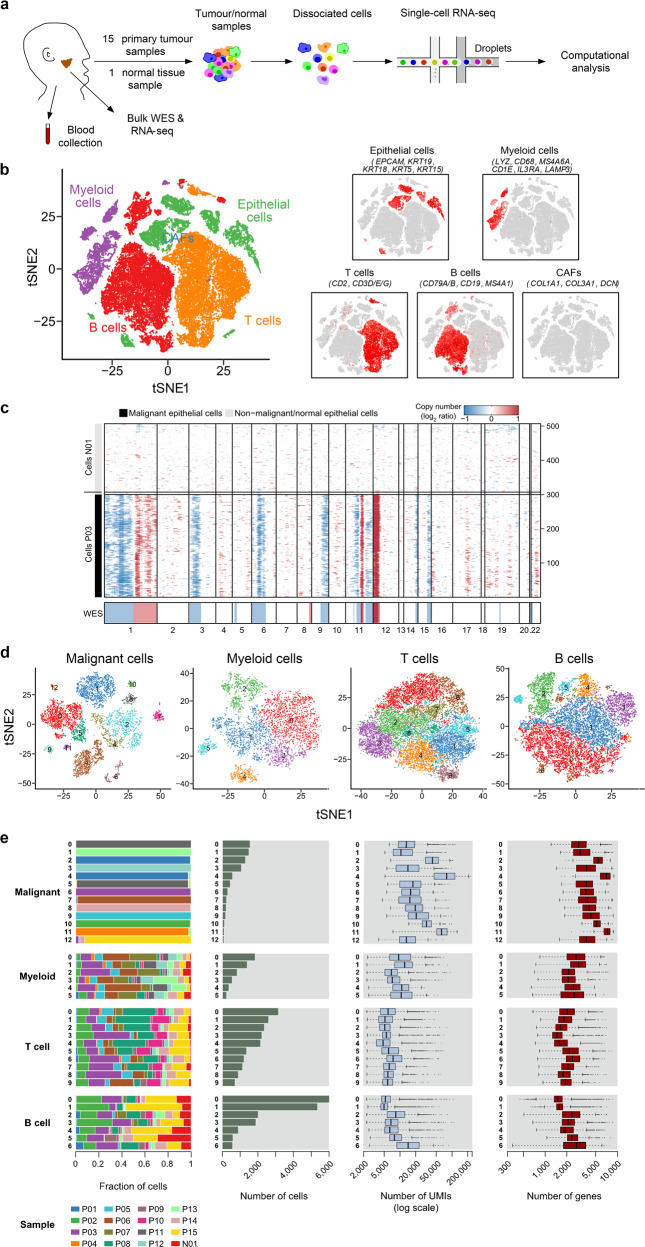
Dissection of the tumor microenvironment in NPC with scRNA-seq. **a** Workflow diagram showing the collection and processing of fresh biopsy samples from 15 primary NPC tumors and one normal tissue for scRNA-seq. **b** t-SNE plots of cells from the 16 samples profiled in this study, with each cell color coded to indicate the associated cell types. The panels on the right show the expression of curated gene sets in the cell types defined in the left panel. **c** Chromosomal landscape of inferred large-scale CNVs distinguishing malignant epithelial cells from non-malignant epithelial cells. The P03 tumor is shown with individual cells (*y*-axis) and chromosomal regions (*x*-axis). Amplifications (red) or deletions (blue) were inferred by averaging expression over 100-gene stretches on the indicated chromosomes. Inferred CNVs are concordant with the calls from WES (bottom). The inferred CNV pattern of the normal epithelial cells from N01 is also shown (top). **d** t-SNE plots showing the subclusters of malignant cells, myeloid cells, T cells, and B cells. **e** Data of the 13 malignant cell subclusters and the 23 immune cell subclusters of 47,866 cells from 16 samples (from left to right): the fraction of cells originating from each patient, the number of cells, and box plots of the number of UMIs and genes (with the box plot center, box, whiskers, and points corresponding to the median, interquartile range, 1.5× interquartile range, and outliers, respectively).

We generated scRNA-seq profiles for primary tumors from 15 patients with treatment-naïve NPC; normal nasopharyngeal epithelial tissue from one patient with chronic nasopharyngitis was also collected (Fig. 1a; Supplementary information, Fig. S1 and Table S1). The fresh biopsies, which were collected during endoscopy, were rapidly digested into single-cell suspensions and analyzed using droplet-based single-cell transcriptome profiles (10× Genomics Chromium system). We also obtained whole-exome sequencing (WES) and bulk RNA-seq data from 12 of the 15 NPC primary tumors (Supplementary information, Table S1).

After quality filtering and doublet removal (see Materials and methods), we profiled a total of 48,584 single cells from the NPC tumor samples and normal tissue sample (Supplementary information, Table S2). Following gene expression normalization, we applied principal component analysis to evaluate variably expressed genes and subsequently used a graph-based clustering method^15,16^ to classify the cells into coherent transcriptional clusters. Then, we annotated the cell clusters by the average expression of curated gene sets and identified immune cells (i.e., myeloid, T/natural killer (NK), and B cells) and cancer-associated fibroblasts (CAFs) in addition to epithelial cells (including malignant and non-malignant cells) (Fig. 1b). No pronounced effects of cell dissociation on gene expression^17^ were noted (Supplementary information, Fig. S2a, b), and the bulk RNA-seq data correlated well with the scRNA-seq data (*r* = 0.71; Supplementary information, Fig. S2c).

We then distinguished malignant from non-malignant cells within the epithelial clusters in two steps. First, as NPC arises from the nasopharyngeal epithelium, we extracted all the epithelial cells based on the initial clustering and cell-type identification described. Second, we inferred large-scale chromosomal copy-number variations (CNVs) in each epithelial cell.^18–20^ These inferred CNVs, which were consistent with the WES data (Fig. 1c), were used to separate the malignant cells from the non-malignant cells with normal karyotypes. We then performed hierarchical clustering of the CNV profiles of the epithelial cells from the normal sample and each of the 15 tumor samples separately^21^; the non-malignant cells of each tumor sample were identified as those in the cluster that predominantly contained cells from the normal sample, while the cells with deletions and amplifications of entire chromosomes in the other cluster were identified as malignant cells. Overall, 7581 malignant cells from 11 tumor samples containing at least 50 malignant cells were identified and retained for further analyses.

Then, we performed normalization, dimensionality reduction and clustering within each major cell type (i.e., malignant, myeloid, T/NK, and B cells) to identify subclusters (see Materials and methods); CAFs were removed from analyses as fewer than 100 cells were detected. This approach revealed a complex TME, containing 13 malignant cell subclusters and 23 different immune cell subclusters (Fig. 1d, e); subsequently we identified signature genes for each of the immune subclusters, successfully developing 17 immune subtype-specific signatures (Supplementary information, Table S3). The high abundance of major immune cell subtypes in NPC was then confirmed by multiplex immunofluorescence analysis (Supplementary information, Fig. S3), especially for the T/NK cells, and the mean percentages of CD4^+^ T, CD8^+^ T, and CD57^+^ NK cells in all cells were 19%, 15% and 2%, respectively. Furthermore, we revealed the disparities in distributions of these cell subtypes within tumors (Supplementary information, Fig. S3c, d). Generally, the immune cells were more enriched in the tumor stroma compared to the tumor epithelial nests, in particular for the CD20^+^ B cells, CD57^+^ NK cells and IL3RA^+^ plasmacytoid dendritic cells (pDCs). Notably, the infiltration of CD8^+^ T cells in tumor epithelial nests was relatively higher than that of other immune cell types, suggesting spatial heterogeneity of different immune cell subtypes within the tumors. It should be noted that few CAFs and endothelial cells were detected in the scRNA-seq, while the mean percentages of these cell types detected by immunofluorescence staining were 4% and 3%, respectively (Supplementary information, Fig. S3b). The differences reflect the well-known disparities in single-cell dissociation efficiency that influence the recovery of individual cell types,^22^ especially for fibroblasts and endothelial cells, which are more deeply embedded in the extracellular matrix and basement membrane than immune cells, and hence are more difficult to dissociate.^13,22^

### Identification of signatures reflecting the intratumoral transcriptional heterogeneity of malignant cells

**Fig. 2:**
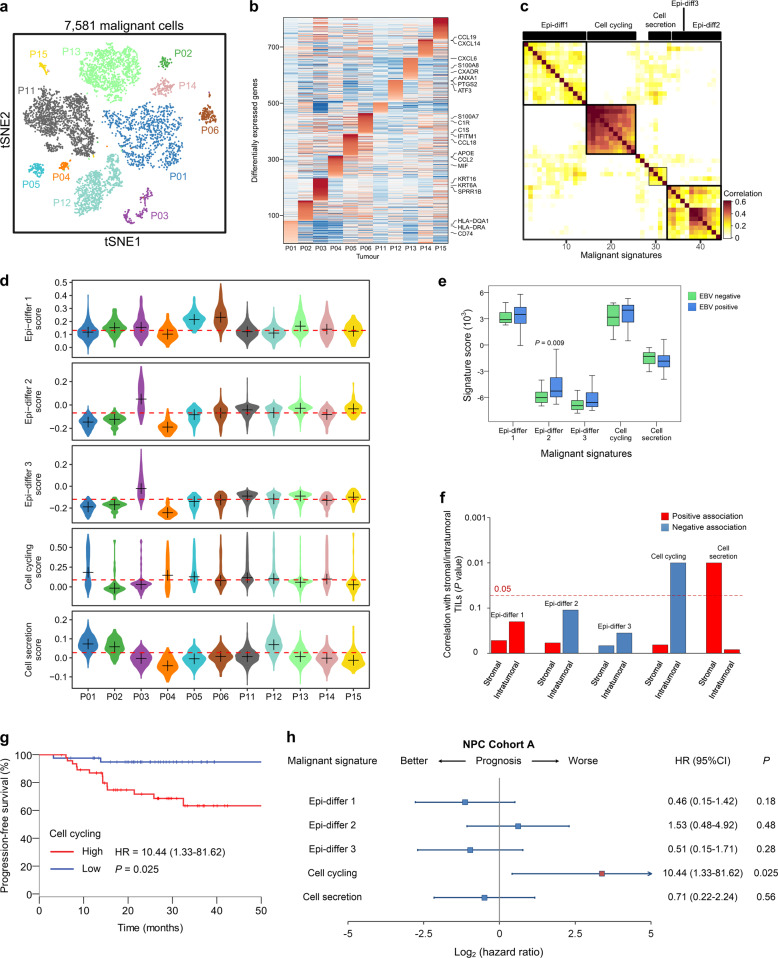
Malignant cell clusters and common malignant signatures revealed in NPC. **a** t-SNE plot of 7581 malignant cells from 11 patients (indicated by colors) reveals tumor-specific clusters. **b** A heatmap shows genes (rows) that are differentially expressed across 11 individual primary tumors (columns). Red: high expression; blue: low expression. Selected genes are highlighted. **c** A heatmap depicts the pairwise correlations of 44 metagenes derived from 11 tumors. Clustering identified five coherent malignant gene expression signatures across the tumors. **d** Each panel (from top to bottom) shows violin plots that depict the scores for one of the five malignant signatures for malignant cells from the 11 tumors. **e** Changes in gene expression for the five malignant signatures in response to different EBV infection statuses (95 positive vs 14 negative, detected by in situ hydridization with the EBV-encoded small RNAs) in NPC Cohort A are shown (*P* values were based on the Wilcoxon rank-sum test). The box plot center corresponds to the median, with the box and whiskers corresponding to the interquartile range and 1.5× interquartile range, respectively. **f** A bar plot shows the direction and statistical significance (*P* values were based on the Spearman correlation test) of the associations between each of the malignant signatures and stromal/intratumoral TILs in NPC Cohort A. **g** Kaplan–Meier curves for progression-free survival in the 88 patients in NPC Cohort A stratified according to high vs low expression of the cell cycling signature. Cox regression HR and 95% CI obtained after correcting for age, sex, smoking history and disease stage are shown; the corresponding Cox regression *P* value is also shown. **h** Prognostic values of the malignant signatures in the 88 patients in NPC Cohort A. Forest plots show HRs (blue/red squares) and CIs (horizontal ranges) derived from Cox regression survival analyses for progression-free survival in multivariable analyses adjusted for age, sex, smoking history and disease stage; the corresponding Cox regression *P* values are also shown. Significant results are indicated with red squares.

Clustering of malignant cells revealed tumor-specific clusters, suggesting a high degree of intertumoral heterogeneity (Fig. 2a). Over 700 genes were preferentially expressed in the individual tumors (Fig. 2b). Differentially expressed genes were enriched within pathways that varied across the tumors, showing significant phenotypic diversity (Supplementary information, Fig. S4). We then applied dimension reduction analyses, specifically non-negative matrix factorization (NMF), and identified a total of 44 metagenes that were preferentially co-expressed by subpopulations of malignant cells across tumors (Supplementary information, Table S4). Then, hierarchical clustering was used to characterize these 44 metagenes into gene expression signatures, and high concordance was shown among five signatures (Fig. 2c; Supplementary information, Table S5).

Of these signatures, the three epithelial differentiation signatures consisted primarily of epithelial genes, such as cytokeratins (e.g., *KRT6*), and small proline-rich proteins (e.g., *SPRR2* and *SPRR3*) (Supplementary information, Table S5). The fourth signature was related to cell cycling, consisting of cell cycle-related genes, and the final signature was enriched for genes associated with cell secretion (Supplementary information, Table S5). The scores for the malignant signatures varied across the malignant cells from different tumors (Fig. 2d). Intriguingly, the signatures were preferentially, but not exclusively, co-expressed by subpopulations of malignant cells (Supplementary information, Fig. S5a). When clustering using only genes from the malignant signatures, the malignant cells were separated into different clusters corresponding mainly to the malignant signatures (Supplementary information, Fig. S5b, c). These results indicated that the malignant signatures we established capture common patterns of intratumoral transcriptional heterogeneity across nasopharyngeal tumors.

### Correlations of malignant signatures with tumor characteristics and survival

Then, we analyzed the correlations between the malignant signatures and clinicopathological features in two independent cohorts with bulk gene expression data: NPC Cohort A (*n* = 113), which was previously described by Zhang and colleagues,^10^ and NPC Cohort B (*n* = 128), which was profiled by an Affymetrix HTA 2.0 microarray at Sun Yat-sen University Cancer Center. Significantly higher Epi-differ 2 scores were observed in EBV-positive tumors than in EBV-negative tumors (Fig. 2e). We then identified genes whose expression was strongly associated with the expression of Epi-differ 2 signature (Supplementary information, Table S6).^23^ Intriguingly, genes related to viral entry into host cell (i.e., *TMPRSS2*, *PVRL4*, *CTSB*, and *SLC20A2*) were revealed to be highly associated with the Epi-differ 2 signature, suggesting their potential roles in promoting epithelial differentiation by regulating EBV infection in NPC. Stromal tumor-infiltrating lymphocytes (TILs) were positively associated with the cell secretion signature (Fig. 2f), suggesting that high expression of the cell secretion signature may promote lymphocyte recruitment to the TME in NPC. In contrast, the negative correlation between the intratumoral TILs and the cell cycling signature may reflect an artifact of the tumor–immune interaction (Fig. 2f), with reduced infiltration of intratumoral TILs hampering the recognition and destruction of mutant tumor cells, in which mutations of G1/S cell cycle transition genes are commonly observed in NPC.^7^ Correlations between the malignant signatures and smoking history and T (tumor) category are also shown (Supplementary information, Fig. S6a). Of note, the Epi-differ 3 signature was positively associated with smoking history as well as the expression of *MALAT1* (*r* = 0.46; *P* < 0.001), which has been reported to mediate lung carcinogenesis induced by cigarette smoke extract.^24^ This observation suggests the potential role of Epi-differ 3-related genes in smoking-mediated epithelial differentiation in NPC.

Furthermore, survival analyses revealed that high cell cycling scores significantly predicted poor survival in NPC Cohort A (Fig. 2g, h). This association with survival were validated in NPC Cohort B (Supplementary information, Fig. S7a), suggesting that the cell cycling signature predicts the aggressiveness of malignant cells in NPC. Additionally, to assess the prognostic role of the cell cycling signature in a different subtype of head and neck cancers, we analyzed the expression of the corresponding signature genes in a head and neck squamous cell carcinoma (HNSCC) cohort using a dataset from The Cancer Genome Atlas (TCGA HNSCC cohort), which comprises 366 HNSCC samples and no NPC samples^25^ (Supplementary information, Table S7). Intriguingly, the cell cycling signature did not predict survival in HNSCC (Supplementary information, Fig. S7b), indicating that this may not be the critical expression program in malignant cells that decides the aggressiveness of HNSCC.

### Novel myeloid cell subtypes and regulators underlying monocyte-macrophage differentiation in NPC

**Fig. 3:**
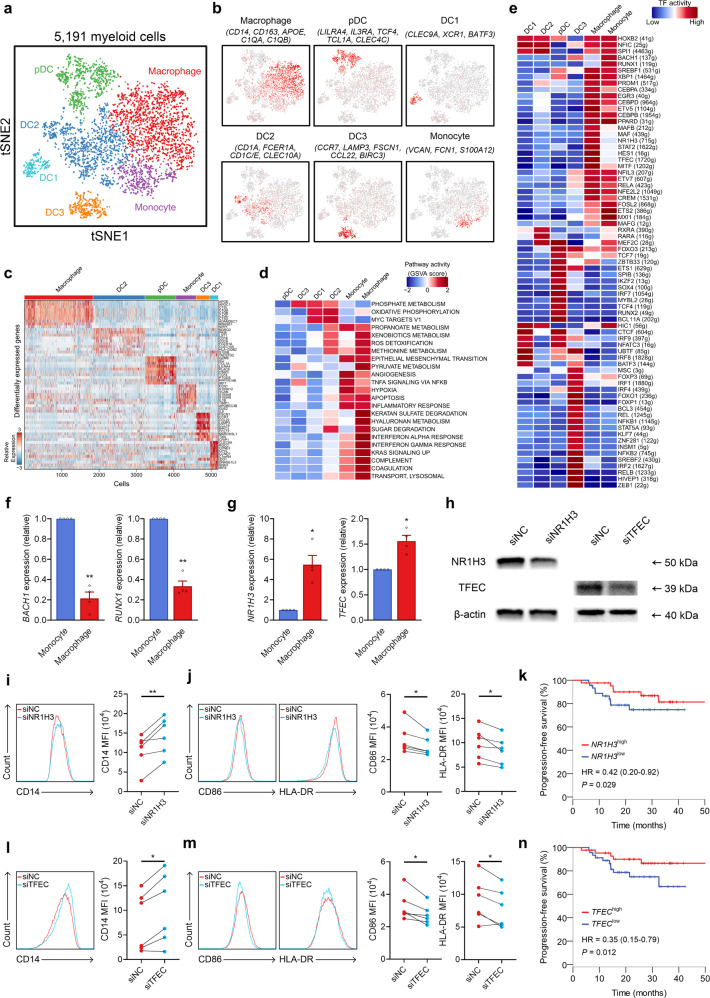
Myeloid cell clusters in NPC. **a** t-SNE plot of 5191 myeloid cells color-coded by their associated clusters. **b** t-SNE plot, color coding for the expression of the marker genes (gray to red) for the indicated cell subtype. **c** Heatmap of genes with differential expression (rows) among the myeloid cell subtypes. **d** Differences in pathway activities scored per cell by GSVA among the different myeloid cell subtypes. The scores of pathways are normalized. **e** Heatmap showing the activity of TFs in each myeloid cell subtypes. The TF activity is scored using AUCell. **f** Expression of *BACH1* and *RUNX1* in primary human monocytes and monocyte-derived macrophages. Data are presented as the means ± SEM of three independent experiments (*n* = 4). **g** Expression of *NR1H3* and *TFEC* in primary human monocytes and monocyte-derived macrophages. Data represent the means ± SEM of two independent experiments (*n* = 4). **h** Primary human monocytes were transfected with negative control (NC), NR1H3-specific, or TFEC-specific siRNAs, followed by immunoblot analysis to determine protein expression of NR1H3 or TFEC. β-actin is the loading control. The experiments were repeated independently for three times with similar results. **i**, **l** Left, representative histograms of CD14 expression levels in siNC and siNR1H3 (*n* = 6 donors, *n* = 4 independent experiments) (**i**), and siTFEC (*n* = 6 donors, *n* = 4 independent experiments) (**l**) monocytes stimulated by M-CSF. Right, expression levels of CD14 in siNC and siNR1H3 (*n* = 6) (**i**), and siTFEC (*n* = 6) (**l**) monocytes stimulated by M-CSF, which was determined by FACS (MFI, mean fluorescent intensity). **j**, **m** Left, representative histograms of CD86 and HLA-DR expression levels in siNC and siNR1H3 (*n* = 6 donors, *n* = 4 independent experiments) (**j**), and siTFEC (*n* = 6 donors, *n* = 4 independent experiments) (**m**) monocytes stimulated by M-CSF. Right, expression levels of CD86 and HLA-DR in siNC and siNR1H3 (*n* = 6) (**j**), and siTFEC (*n* = 6) (**m**) monocytes stimulated by M-CSF, which was determined by FACS (MFI). **k**, **n** Kaplan–Meier curves showing progression-free survival in the 88 patients in NPC Cohort A stratified according to high vs low expression of *NR1H3* (**k**) and *TFEC* (**n**). Cox regression HRs and 95% CIs obtained after correcting for age, sex, smoking history and disease stage are shown; the corresponding Cox regression *P* values are also shown. *P < 0.05, **P < 0.01 (paired Student’s *t*-test).

In total, 5191 myeloid cells were clustered into six separate subsets (Fig. 3a): macrophages, monocytes, pDCs, *CLEC9A*^+^ DCs (DC1), *CD1C*^+^ DCs (DC2), and *CCR7*^+^ DCs (DC3) (Fig. 3b). Notably, DC1 expressed many of the classic markers of conventional type 1 DCs (cDC1), including *CLEC9A*, *IRF8*, and *IDO1* (Fig. 3c).^26^ cDC1 constitute the key DC subtype responsible for cross-priming antitumor CD8^+^ T cells and are critical in antitumor immunity.^27^ DC3 expressed markers, such as *CCR7*, *LAMP3*, *FSCN1*, and *CCL19*, that have been associated with DC activation. Of these markers, CCL19 is known to attract naïve T cells as well as other DCs by interacting with CCR7 (Fig. 3c).^28,29^ DC2 preferentially expressed a series of classic marker genes of conventional type 2 DCs (cDC2), including *CD1E*, *FCER1A* and *CLEC10A* (Fig. 3c). Immunofluorescence confirmed the presence of distinct populations of DC1 and DC3 cells in NPC tissue samples (Supplementary information, Fig. S8). *GZMB*, which encodes the pro-apoptotic enzyme granzyme B, was highly expressed in pDCs (Fig. 3c), suggesting that the cells in this population may exhibit GZMB-dependent cytotoxicity.^30^ Interestingly, a relatively high proportion of pDCs was observed in our scRNA-seq data (2% in all cells, and 16% in myeloid cells) compared to other cancer types (1%, 3%, and 10% in myeloid cells for lung cancer,^13^ breast cancer,^31^ and HNSCC,^32^ respectively). This observation was validated by immunofluorescence analysis showing a mean percentage of 3% in all cells for pDCs, with a highly notable aggregation in the tumor stroma (Supplementary information, Fig. S3d).

We further characterized the functions of different myeloid cell subtypes by comparing pathway activities. Although macrophages and monocytes exhibited some common activated pathways, pathways involved in interferon (IFN)-α and IFN-γ responses were relatively upregulated in the macrophages, whereas angiogenesis-, NF-κB-mediated TNF-α signaling-, and hypoxia-related pathways were upregulated in the monocytes (Fig. 3d). These results indicate the potential tumor-promoting features of monocytes in NPC,^33^ consistent with their high expression of *S100A8* and *S100A9* (Fig. 3c).^34^ We then applied Single-Cell Regulatory Network Inference And Clustering (SCENIC) analysis^35^ to correlate transcription factors (TFs) with gene expression differences among cell types. This analysis identified a set of TFs implicated in the biology of different myeloid cell subtypes in NPC (Fig. 3e). Interestingly, macrophages and monocytes shared similar expression patterns for many TFs (Fig. 3e), suggesting that the macrophages may be derived from monocytes recruited to the NPC TME.^36^ Of note, the expression of genes regulated by BACH1 and RUNX1 was specifically upregulated in monocytes, whereas expression of NR1H3 and TFEC was prominent in macrophages (Fig. 3e). Next, we stimulated primary human monocytes with M-CSF to obtain macrophages, and observed significantly decreased expression of *BACH1* and *RUNX1*, and increased expression of *NR1H3* and *TFEC* in the monocyte-derived macrophages (Fig. 3f, g). Though previous reports have suggested the function of BACH1 and RUNX1 in promoting monocyte development,^37,38^ the roles of NR1H3 and TFEC remain unknown in the monocyte-to-macrophage differentiation process. To investigate the function of NR1H3 and TFEC, we subsequently transfected primary human monocytes with negative control (NC) or NR1H3/TFEC-specific siRNAs (Fig. 3h). After being triggered by M-CSF, the expression of CD14 was significantly higher in siNR1H3 (Fig. 3i) or siTFEC (Fig. 3l) cells than in siNC cells; the induction of CD86 and HLA-DR was also impaired in siNR1H3 or siTFEC cells (Fig. 3j, m). These results support that NR1H3 and TFEC facilitate the differentiation and maturation of monocyte-derived macrophages, suggesting that these regulators may promote antitumor immunity in NPC. Interestingly, further survival analyses revealed the association between high expression levels of *NR1H3* and *TFEC* and improved outcomes in NPC patients (Fig. 3k, n). Our data provide a resource for further investigation of regulators underlying myeloid diversity in NPC.

### T/NK cell phenotypes suggest regulators modulating antitumor immune response in NPC

**Fig. 4:**
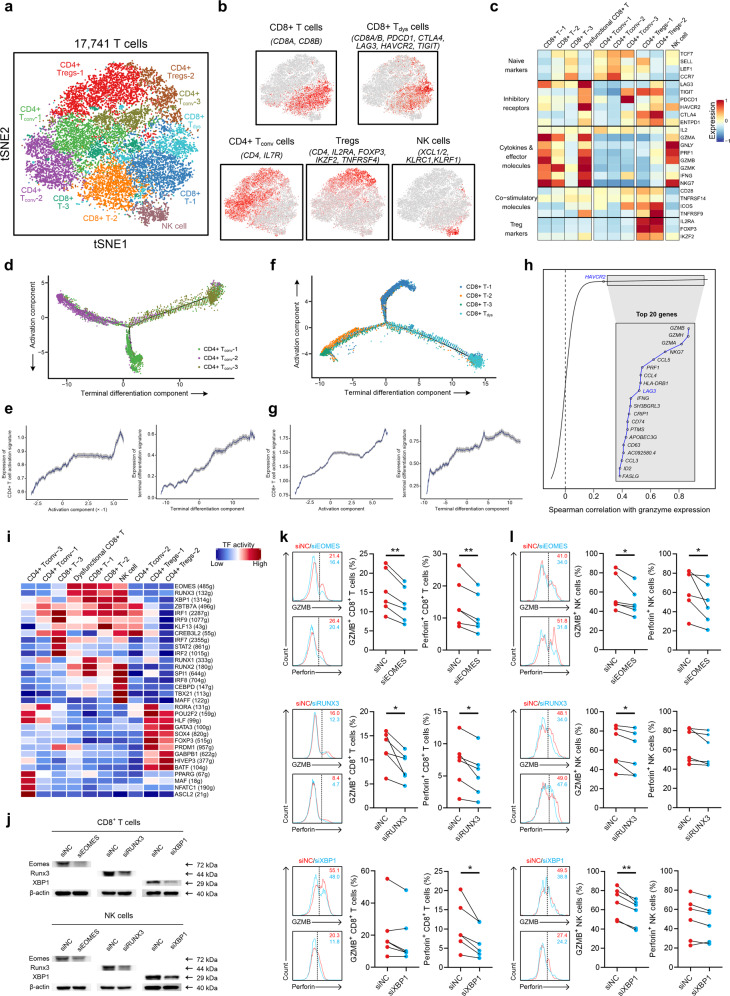
T/NK cell clusters in NPC. **a** t-SNE plot showing 10 clusters of 17,263 T/NK cells (indicated by colors). **b** t-SNE plot, color coding for the expression of the marker genes (gray to red) for the indicated cell subtypes. **c** Average expression of selected T cell function-associated genes of naïve markers, inhibitory receptors, cytokines and effector molecules, co-stimulatory molecules, and Treg markers in each cell cluster. **d** Potential developmental trajectory of CD4^+^ T cells (*n* = 5694) inferred by analysis with Monocle 2. Arrows show the increasing directions of certain CD4^+^ T cell properties annotated with the signatures shown in **e**. **e** Traceplots of (left) CD4^+^ T cell activation signature along activation component and (right) terminal differentiation signature along terminal differentiation component for the CD4^+^ T cells. Cells are projected along the component, with the blue line indicating the moving average of the expression of signatures (a sliding window of length equal to 5% of the total number of CD4^+^ T cells was used), and the shaded area displaying SEM. Signatures used are presented in Supplementary information, Table S9. **f** Potential developmental trajectory of CD8^+^ T cells (*n* = 6975) inferred by analysis with Monocle 2. Arrows show the increasing directions of certain CD8^+^ T cell properties annotated with the signatures shown in **g**. **g** Traceplots (as in **e**) of (left) CD8^+^ T cell activation signature along activation component and (right) terminal differentiation signature along terminal differentiation component for the CD8^+^ T cells. Signatures used are presented in Supplementary information, Table S9. **h** Spearman correlation between the activity of CD8^+^ T cells (*n* = 6975), as measured by average granzyme expression (*GZMA*, *GZMB* and *GZMH*), and the expression of CD8^+^ T cell-specific genes. Genes encoding known immune checkpoint molecules are highlighted in blue. CD4^+^ T_conv_, conventional CD4^+^ T cell; CD8^+^ T_dys_, dysfunctional CD8^+^ T cell; NK, natural killer. **i** Heatmap showing the activity of TFs in each T/NK cell subtype. The TF activity is scored using AUCell. **j** Peripheral CD8^+^ T cells and NK cells were transfected with negative control (NC), EOMES-specific, RUNX3-specific, or XBP1-specific siRNAs, followed by immunoblot analysis to determine protein expression of Eomes, Runx3, or XBP1. β-actin is the loading control. The experiments were repeated independently for three times with similar results. **k** Left, representative histograms depicting the expression of GZMB and perforin on peripheral CD8^+^ T cells transfected with NC and EOMES-specific (*n* = 6 donors, *n* = 5 independent experiments), RUNX3-specific (*n* = 6 donors, *n* = 6 independent experiments), and XBP1-specific siRNAs (*n* = 6 donors, *n* = 5 independent experiments). Right, percentage of GZMB^+^ or perforin^+^ cells in siNC and siEOMES (*n* = 6), siRUNX3 (*n* = 6), and siXBP1 (*n* = 6) CD8^+^ T cells. **l** Left, representative histograms depicting the expression of GZMB and perforin on peripheral NK cells transfected with NC and EOMES-specific (*n* = 6 donors, *n* = 5 independent experiments), RUNX3-specific (*n* = 6 donors, *n* = 5 independent experiments), and XBP1-specific siRNAs (*n* = 6 donors, *n* = 5 independent experiments). Right, percentage of GZMB^+^ or perforin^+^ cells in siNC and siEOMES (*n* = 6), siRUNX3 (*n* = 6), and siXBP1 (*n* = 6) NK cells. *P < 0.05, **P < 0.01 (paired Student’s *t*-test).

T/NK cells (*n* = 17,741) represented the most prevalent cell type in NPC. Reclustering revealed 10 clusters: CD8^+^ T-1, CD8^+^ T-2, CD8^+^ T-3, dysfunctional T (CD8^+^ T_dysfunctional_) cells, conventional CD4^+^ T (CD4^+^ T_conv_)-1, CD4^+^ T_conv_-2, CD4^+^ T_conv_-3 cells, regulatory T cells (Tregs)-1, Tregs-2, and NK cells (Fig. 4a, b). Next, we compared the expression levels of selected T cell function-associated genes among the different T cell subtypes (Fig. 4c). Among the CD8^+^ T-1, CD8^+^ T-2 and CD8^+^ T-3 subtypes, high expression of cytokines and effector molecules was exhibited in CD8^+^ T-1, followed by CD8^+^ T-2 and CD8^+^ T-3 (Fig. 4c), suggesting that CD8^+^ T-1 represented the main cytotoxic population in NPC. CD4^+^ T_conv_-2 represented naïve T cells, while exhaustion markers such as *PDCD1* and *TIGIT* were highly expressed in CD4^+^ T_conv_-3 (Fig. 4c). Monocle trajectory analysis of CD4^+^ T cells inferred a differentiation trajectory that mainly began with the CD4^+^ T_conv_-2 naïve cluster and bifurcated into either the CD4^+^ T_conv_-1 activation cluster or the CD4^+^ T_conv_-3 dysfunctional/terminal differentiation cluster (Fig. 4d, e). For conventional CD8^+^ T cells, the differentiation trajectory also exhibited a branched structure, starting with CD8^+^ T-3 and T-2 that bifurcated into either a cytotoxic/activation lineage (mainly CD8^+^ T-1) or a dysfunctional lineage (mainly CD8^+^ T_dysfunctional_) (Fig. 4f, g). Notably, we observed high expression of immune checkpoint molecules, except CTLA4, in the CD8^+^ T_dysfunctional_ cells, and LAG-3 and HAVCR2 were the most prominently expressed molecules (Fig. 4c, h). Of note, the expression levels of these immune checkpoint molecules were generally comparable in CD8^+^ T_dysfunctional_ cells in HNSCC^12^ (data not shown), suggesting distinctive features of dysfunctional T cells in NPC TME compared to those in other head and neck cancers. Intriguingly, exhaustion gene expression was highly associated with the expression of cytotoxicity markers (Fig. 4c), as *LAG3* and *HAVCR2* were found to be correlated with T cell activity, which was measured by the mean expression of granzymes (*GZMA*, *GZMB* and *GZMH*) (Fig. 4h). The expression of *LAG3* and *HAVCR2* also exhibited significant positive associations with intratumoral TILs (Supplementary information, Fig. S9a), and immunofluorescence analysis confirmed the higher percentage of LAG-3^+^ and HAVCR2^*+*^ CD8^+^ T cells in the total CD8^+^ T cells in tumor epithelial nests compared with those in the tumor stroma (Supplementary information, Fig. S9b). A trend of better survival was also shown for higher expression of *LAG3* and *HAVCR2* (Supplementary information, Fig. S9c). These results reflected an activation-dependent exhaustion expression program^13,14^ especially for LAG-3 and HAVCR2 in NPC, which represent potentially appealing checkpoint molecules for therapy.

SCENIC analysis revealed potential TFs underlying the regulation across subtypes (Fig. 4i). For example, NFATC1 was identified as a candidate TF underlying the gene expression differences in CD4^+^ T_conv_-3 (Fig. 4i). NFATC1 is reported to regulate PD-1 expression following T cell activation,^39^ which is consistent with the upregulated PD-1 expression observed in CD4^+^ T_conv_-3 and implicates NFATC1 as a potential regulator of CD4^+^ T_conv_ cells in their way to become dysfunctional in NPC. Interestingly, NK cells shared upregulated expression patterns of TFs such as EOMES, RUNX3, and XBP1 with cytotoxic CD8^+^ T cell subtypes (Fig. 4i), indicating the potential roles of these TFs in regulating the cytotoxic activity of both CD8^+^ T and NK cells in NPC. Previous studies have suggested that EOMES and RUNX3 contribute to the development and homeostasis of effector and memory T cells as well as NK cell differentiation.^40–42^ Nevertheless, the role of XBP1 in regulating cytotoxic lymphocytes is less well understood,^43–45^ and the effects of these regulators in immune responses have not been addressed in NPC. To validate the SCENIC results, we isolated CD8^+^ T cells and NK cells from the NPC tissue samples by flow cytometry (FACS), and identified a significantly higher percentage of cytotoxic GZMB^+^ or perforin^+^ phenotypes in the EOMES^+^, RUNX3^+^, and XBP1^+^ CD8^+^ T cells and NK cells (Supplementary information, Fig. S10a, b). Furthermore, reducing the expression of EOMES, RUNX3, and XBP1 in primary human CD8^+^ T cells and NK cells by transfection with corresponding siRNAs also downregulated the expression of GZMB or perforin compared with siNC cells (Fig. 4j–l), suggesting their function in regulating immune cytolytic activity. These data reveal compelling TFs enhancing the cytotoxicity of both CD8^+^ T cells and NK cells in NPC.

### Diverse B cell subtypes identified in NPC

**Fig. 5:**
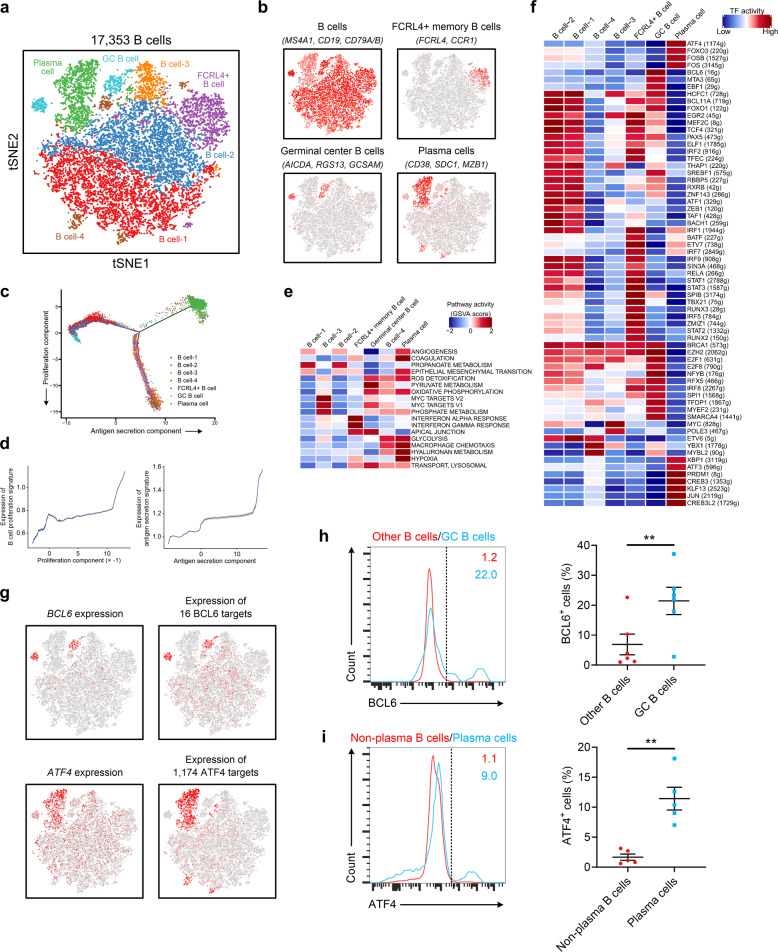
B cell clusters in NPC. **a** t-SNE plot showing 10 clusters of 17,353 B cells (indicated by colors). **b** t-SNE plot, color coding for the expression of the marker genes (gray to red) for the indicated cell subtypes. **c** Potential developmental trajectory of B cells (*n* = 17,353) inferred by analysis with Monocle 2. Arrows show the increasing directions of certain B cell properties annotated with the signatures shown in **d**. **d** Traceplots of (left) B cell proliferation signature along proliferation component and (right) antigen secretion signature along antigen secretion component for the B cells. Cells are projected along the component, with the blue line indicating the moving average of the expression of signatures (a sliding window of length equal to 5% of the total number of B cells was used), and the shaded area displaying SEM. Signatures used are presented in Supplementary information, Table S9. **e** Comparison of pathway activities (calculated based on GSVA) among different B cell subtypes. The scores of pathways are normalized. **f** Heatmap showing the activity of TFs in each B cell subtypes. The TF activity is scored using AUCell. **g** t-SNE plots of B cells color coded according to the expression of *BCL6* and *ATF4* or the AUC of the estimated regulon activity of these transcription factors, which corresponded to the degree of expression regulation of their target genes. **h** Left, FACS analysis of BCL6 expression in GC and other B cells from tumor tissues. The results represent five independent experiments (*n* = 6 donors). Right, association between GC B cells and BCL6^+^ B cells (*n* = 6) from tumor tissues. **P < 0.01 (paired Student’s *t*-test). Error bars, SEM. **i** Left, FACS analysis of ATF4 expression in plasma cells and non-plasma B cells from tumor tissues. The results represent four independent experiments (*n* = 5 donors). Right, association between plasma cells and ATF4^+^ B cells (*n* = 5) from tumor tissues. *P < 0.05 (paired Student’s *t*-test). Error bars, SEM.

We detected 17,353 B cells that could be divided into seven clusters (Fig. 5a, b). Of these clusters, one consisted of rarely reported FCRL4^+^ memory B cells, in which elevated CCR1 expression supports the homing of these cells to inflamed tissues.^46^ One cluster comprised germinal center (GC) B cells, whereas another comprised plasma cells; it has been reported that GC B cells with relatively high affinity could be directed to become plasma cells.^47^

Then, we analyzed all B cells to infer the potential developmental trajectory. The results showed that plasma cells congregated at the furthest terminus of the antigen secretion components, whereas FCRL4^+^ B cell/B cell-3/B cell-4 clusters were enriched at the end of the proliferation components, suggesting trajectories of functional divergence among these cell types (Fig. 5c, d). Differences in pathway activities and TFs among the different B cell subtypes are shown in Fig. 5e, f, respectively. GC B cells exhibited an activation of the pyruvate metabolism and MYC pathways, while the glycolysis pathway was upregulated in plasma cells (Fig. 5e). In addition, high activities of macrophage chemotaxis pathways were also detected in plasma cells, suggesting the potential roles of these pathways in promoting macrophage recruitment to the TME in NPC (Fig. 5e). Notably, the expression of genes regulated by TFs such as BCL6 and ATF4 was specifically upregulated in GC B cells and plasma cells, respectively (Fig. 5f, g). BCL6, which has been reported to be critical for GC B cell development, reactivates the B cell transcriptional program to enhance responses to external stimuli,^48^ whereas ATF4 plays a role in immunoglobulin production.^49^ These transcriptional phenotypes were confirmed in the NPC tissue samples by FACS (Fig. 5h, i). These results identified candidate regulators underlying the gene expression differences between GC B cells and plasma cells in NPC.

### Complex intercellular communication networks in the NPC TME

**Fig. 6:**
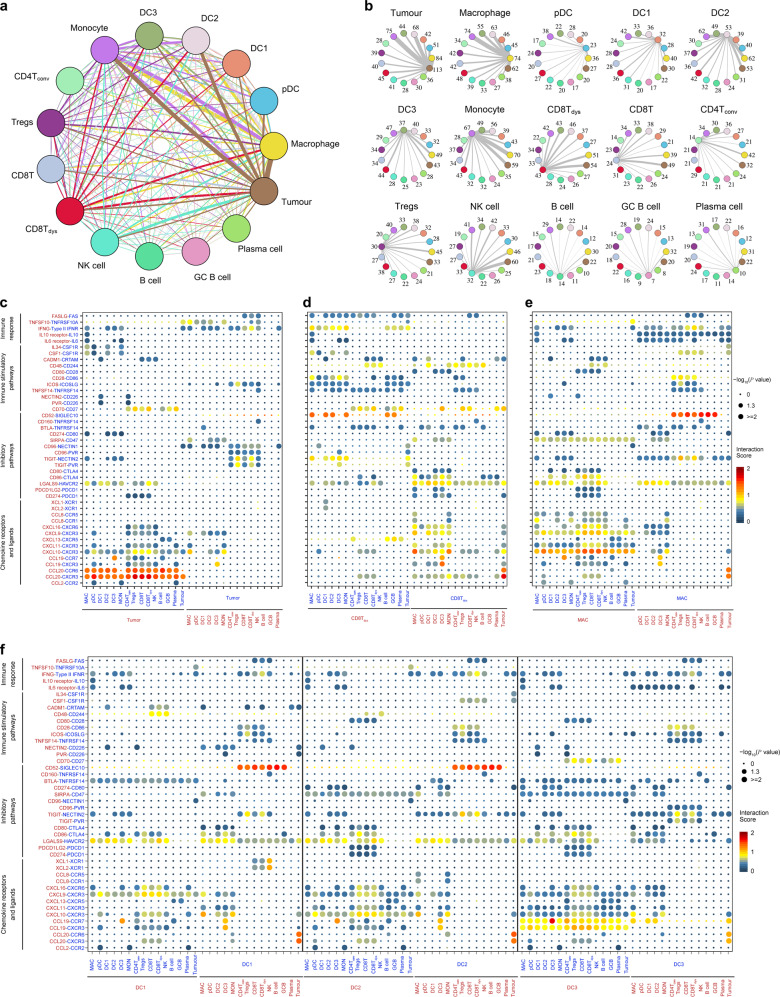
The dense network and multiple regulatory immune responses in the TME of NPC. **a** Capacity for intercellular communication between malignant cells and immune cells. Each line color indicates the ligands expressed by the cell population represented in the same color (labeled). The lines connect to the cell types that express the cognate receptors. The line thickness is proportional to the number of ligands when cognate receptors are present in the recipient cell type. The loops indicate autocrine circuits. The map quantifies potential communication but does not account for the anatomical locations or boundaries of the cell types. **b** Detailed view of the ligands expressed by each major cell type and the cells expressing the cognate receptors primed to receive the signal. Numbers indicate the quantity of ligand–receptor pairs for each intercellular link. **c**–**f** Overview of selected ligand–receptor interactions of tumor cells (**c**), dysfunctional CD8^+^ T cells (**d**), macrophages (**e**), and the three types of DCs (**f**, DC1, DC2, and DC3). *P* values are indicated by circle size, with the scale to the right (permutation test). The means of the average expression levels of interacting molecule 1 in cluster 1 and interacting molecule 2 in cluster 2 are indicated by color. Assays were carried out at the mRNA level but were used to extrapolate protein interactions. CD4T_conv_, conventional CD4^+^ T cell; CD8T, CD8^+^ T cell; CD8T_dys_, dysfunctional CD8^+^ T cell; DC, dendritic cell; GCB, germinal center B cell; MAC, macrophage; MON, monocyte.

Next, we used CellPhoneDB^50^ to identify ligand–receptor pairs and molecular interactions among the major cell types (Fig. 6a). Broadcast ligands for which cognate receptors were detected demonstrated extensive communication between tumor and immune cells (Fig. 6a, b).

Notably, tumor cells expressed relatively high levels of chemokines (e.g., CCL20, CCL19, and CXCL10), while the corresponding receptors were widely expressed in immune cells, suggesting that these chemokines play significant roles in enhancing immune cell infiltration into NPC tumor tissue (Fig. 6c). Inhibitory interactions between tumor cells and T cells were commonly observed. In addition to CD274–PDCD1, other receptor–ligand pairs that have rarely been reported in NPC (e.g., NECTIN1–CD96, NECTIN2–TIGIT, PVR–CD96 and LGALS9–HAVCR2) were also identified (Fig. 6c, d). Specifically, we evaluated the interactions of LAG-3 and its ligands between T cells and tumor cells (Supplementary information, Fig. S11a). The previously reported FGL1–LAG-3 interaction^51^ was not obvious in NPC, primarily due to the low expression of *FGL1* in the tumor cells (Supplementary information, Fig. S11b); MHC-II was predominantly responsible for the inhibitory function of LAG-3 induced by the interaction between dysfunctional T cells and tumor cells. We also identified putative communications between tumor cells and macrophages, such as the inhibitory or stimulatory interactions produced by CD47–SIRPA and CSF1–CSF1R (Fig. 6c, e).^52^

In addition, we identified specific ligand–receptor complexes predicted to regulate multiple immunomodulatory pathways coordinated by diverse subtypes of immune cells (Fig. 6d–f). High chemokine expression was detected in macrophages, DC1, DC2 and DC3, especially in macrophages and DC3, indicating their important roles in recruiting diverse types of immune cells (Fig. 6f). Interestingly, inhibitory communications between DC3 and T cells were also widespread (Fig. 6f), corresponding to the recent study suggesting this DC phenotype to be the most active immune-regulators of lymphocytes.^53^ Of note, DC1 expressed relatively high levels of *XCR1*, and the ligands of *XCR1* (*XCL1* and *XCL2*) were overexpressed by NK cells (Fig. 6f), suggesting the presence of functional interactions between NK cells and DC1. NK cells mediated the recruitment of DC1 to the TME of NPC, which is consistent with previous reports in a mouse model.^54^

### Correlations of immune subtype-specific signatures with survival

**Fig. 7:**
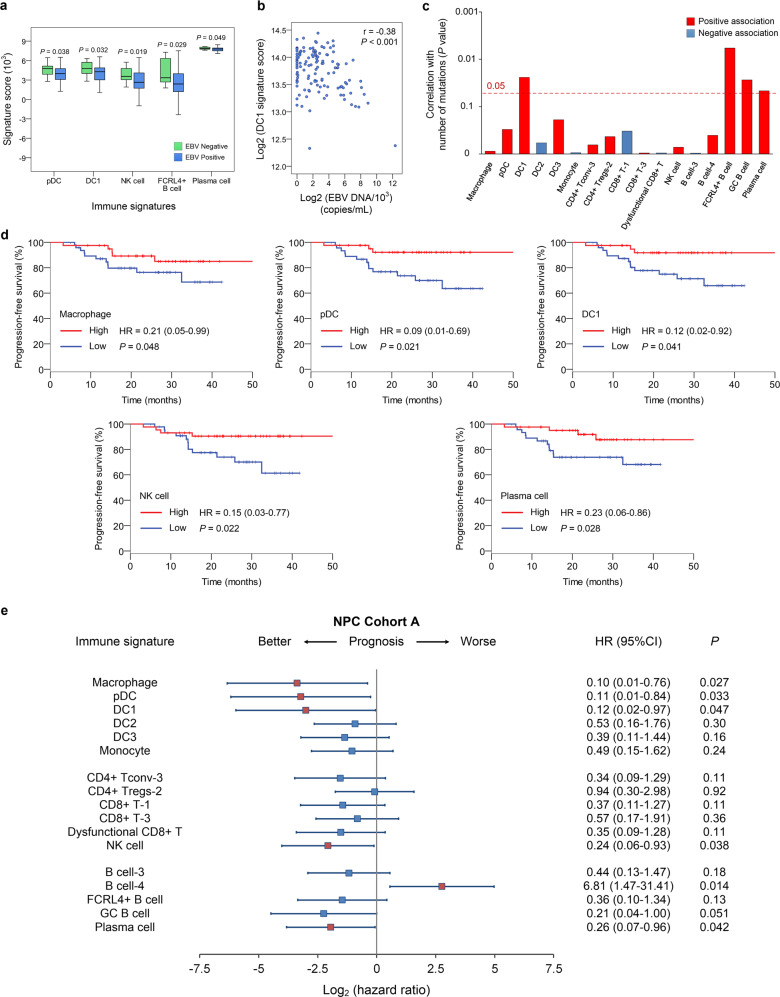
Correlations of immune subtype-specific signatures with clinicopathological features and survival in NPC. **a** Changes in gene expression for the indicated five immune signatures (pDC, DC1, NK, FCRL4^+^ B and plasma cells) with significant associations with the EBV infection status (95 positive vs 14 negative, detected by in situ hydridization with the EBV-encoded small RNAs) in NPC Cohort A. The box plot center corresponds to the median, with the box and whiskers corresponding to the interquartile range and 1.5× interquartile range, respectively. *P* values were based on the Wilcoxon rank-sum test. **b** Density dot plot and Pearson’s correlation analysis (*r*) of the gene expression for the DC1 signature and EBV DNA level in the NPC Cohort B (*n* = 128). **c** Bar plot showing the direction and statistical significance (*P* values were based on the Spearman correlation test) of the association between each of the immune cell subtypes and the number of mutations in NPC Cohort A. Significant associations are shown for the immune signatures DC1 and FCRL4^+^ B, GC B and plasma cells, which were positively correlated with the mutational burden. **d** Kaplan–Meier survival curves for progression-free survival in the 88 patients in NPC Cohort A stratified according to high vs low expression of six immune signatures (macrophage, pDC, DC1, NK cell, and plasma cell). Cox regression HRs and 95% CIs obtained after correcting for age, sex, smoking history and disease stage are shown; the corresponding Cox regression *P* values are also shown. **e** Prognostic values of immune signatures in the 88 patients in NPC Cohort A. Forest plots show HRs (blue/red squares) and CIs (horizontal ranges) derived from Cox regression survival analyses for progression-free survival in multivariable analyses adjusted for age, sex, smoking history and disease stage; the corresponding Cox regression *P* values are also shown. Significant results are indicated by red squares.

Finally, we correlated the 17 immune subtype-specific signatures (Supplementary information, Table S3) derived from the 23 immune cell subtypes with clinicopathological features of NPC patients. Five immune signatures (i.e., pDC, DC1, NK cells, and FCRL4^+^ and plasma B cells) were associated with EBV infection, with all showing significantly lower scores in EBV-positive tumors than in EBV-negative tumors (Fig. 7a). We further explored their correlations with plasma EBV DNA concentrations. *CLEC9A*^+^ DC1 showed a significantly negative correlation with the EBV DNA level (*r* = −0.38; *P* < 0.001), suggesting that DC1 may be affected by active EBV replication (Fig. 7b). CLEC9A is reported to induce antitumor responses^55,56^ and modulate cytotoxic T lymphocyte responses to virus infection in mice^57,58^; however, the mechanisms underlying the influence of EBV infection on the *CLEC9A*^+^ DC-related immune response in NPC remain to be elucidated. Although DC1, FCRL4^+^ B, GC B and plasma cells were significantly associated with a relatively high number of mutations, most immune cell subtypes generally correlated poorly with mutational load (Fig. 7c), probably due to the overall low rate of non-synonymous mutations in NPC.^7,9^ With respect to tumor staging, a general reduction in many immune signatures was shown in patients with relatively high T and N categories and clinical stages (Supplementary information, Fig. S6b).

Subsequently, we explored the prognostic roles of these immune signatures in NPC. Generally, a consistent association between high scores for different immune signatures and improved survival was observed in NPC (Fig. 7d, e; Supplementary information, Fig. S7a). Importantly, positive associations were significant between survival and the signatures of macrophages, pDCs, DC1, NK cells, and plasma cells in NPC Cohort A (Fig. 7d, e). Furthermore, these associations were also evident in NPC Cohort B (Supplementary information, Fig. S7a), validating their reliability as prognostic biomarkers in NPC. Next, we assessed the prognostic value of these five immune signatures (Supplementary information, Table S7) in the TCGA HNSCC cohort. As *CLEC9A*^*+*^ DC1 was not identified in HNSCC studies, the NPC DC1 signature was used instead to infer its prognostic value in HNSCC. No credible prognostic values were identified for the immune signatures in HNSCC (Supplementary information, Fig. S7b), reflecting a difference in the prognostic value of these signatures between NPC and HNSCC.

## Discussion

Through revealing the cellular heterogeneity of the TME, we established the intratumoral and immune subtype-specific signatures in NPC. High scores for the cell cycling signature were associated with poor survival, suggesting that this signature may reflect a highly proliferative and aggressive status in malignant cells. Importantly, high expression of the signatures of macrophages, pDCs, DC1, NK cells, and plasma cells significantly predicted a favorable prognosis in NPC. In this study, we observed high expression of CXC chemokine ligands, such as *CXCL9* and *CXCL10*, that recruit CXCR3^+^ NK and CD8^+^ T cells into the TME, and upregulated activity of the IFN-α and IFN-γ response pathways in macrophages, suggesting potential antitumor capacity of these macrophages in NPC.^59^ The role of pDCs in immune regulation has been extensively debated as they tend to be tolerogenic and are associated with worse clinical outcomes in certain types of cancer, while they also participate in the priming of immunogenic adaptive responses.^60^ The favorable prognostic value of pDCs in NPC suggests the potential role of these cells in inducing antitumor immune responses in this disease. Of note, the signatures did not show a pronounced prognostic value in HNSCC patients. The differences in the TME between NPC and other head and neck cancers may account for the differences in the prognostic value of the signatures. In HNSCC, for example, the approximate proportions of DCs and B cells in immune cells are ~3% and ~10%, respectively,^12,32^ but their proportions in NPC according to our study are ~7% and ~40%, respectively. It should be noted that *CLEC9A*^*+*^ DC1 have not been reported previously in HNSCC, although the specificity of this population in NPC over other HNSCC merits further exploration. As immune cells are distributed unevenly within tumors, future studies with emerging technologies, such as spatial transcriptomics, are warranted to comprehensively elucidate the spatial heterogeneity of NPC TME.

The distinctive features of the TME may provide a foundation for the design of therapies targetting NPC. For instance, analyses of myeloid cells revealed compelling TFs regulating the monocyte-to-macrophage differentiation. Similarly, SCENIC analysis in CD8^+^ T cells and NK cells identified regulators that can enhance their cytotoxicity in NPC. This resource would help deepen our understanding of immune cell diversity and provide directions for future exploration of the transcriptional regulators in immune cell biology. Our data also suggest potential immunotherapeutic targets that may be used in combination with PD-1/PD-L1 blockade to further improve response rates in NPC.^61,62^ LAG-3 and HAVCR2, which have rarely been studied in NPC to date, were highlighted as the most prominent immune checkpoint molecules in CD8^+^ T_dysfunctional_ in NPC. Moreover, the cell–cell interactions suggest that tumor cells communicate extensively with immune cells and that inflammatory and adaptive immune responses are mediated by various immunomodulatory molecules (e.g., TIGIT, CD96, CD47, CSF1R, and XCL1/2-XCR1) that have rarely been studied before in NPC.

In summary, to the best of our knowledge, this work represents a unique resource providing a comprehensive single-cell transcriptome atlas of the multicellular ecosystem of NPC TME. This study lays a new foundation for the development of precision therapies in NPC.

## Materials and methods

### Patient samples

Fifteen patients who were pathologically diagnosed with non-keratinizing or keratinizing NPC were enrolled in this study; normal nasopharyngeal epithelial tissue from one patient with chronic nasopharyngitis was also collected as a control (Fig. 1a). All patients were treatment naïve, and their clinical characteristics are summarized in Supplementary information, Table S1. For 12 of the 15 NPC samples, bulk WES and RNA-seq data were also obtained; matched peripheral blood was collected. Fresh sterile tumor biopsies were collected from the primary site by endoscopy and were initially divided into three segments for the 12 NPC samples, of which two small fragments were immediately transferred into RNA-protect (Qiagen, Hilden, Germany) for bulk RNA and DNA isolation, and the third fragment was fresh-processed for scRNA-seq. The remaining 3 samples were not divided; they were all fresh-processed for scRNA-seq. All clinical samples were collected at Sun Yat-sen University Cancer Center. Written informed consent was obtained from all participants, and ethical approval was obtained from the Institutional Review Board of Sun Yat-sen University Cancer Center (B2016-048-01), in accordance with the Declaration of Helsinki.

### Preparation of single-cell suspensions

The samples for scRNA-seq were washed with phosphate-buffered saline (PBS; Thermo Fisher Scientific, Waltham, MA, USA), placed on ice, cut into small pieces (< 1 mm^3^) and transferred to 5 mL Dulbecco’s modified Eagle’s medium (DMEM; Thermo Fisher Scientific) containing collagenase IV (1 μg/mL) (Thermo Fisher Scientific). The samples were incubated for 40 min at 37 °C with manual shaking every 10 min and then filtered twice using a 40-µm nylon mesh (Thermo Fisher Scientific). Following centrifugation (500 × *g*, 4 °C, and 5 min), the supernatant was decanted and discarded before the cell pellet was resuspended in 1 mL fetal bovine serum (FBS; containing 10% DMSO) and transferred to cryogenic vials. Following a 5-min incubation at room temperature, the samples were centrifuged (120 × *g*, 4 °C, 5 min) using a swing-out rotor. The samples were then resuspended in PBS containing 0.08% bovine serum albumin (BSA; Sigma) and filtered through 40-µm cell strainers (BD) using wide-bore 1-mL low-retention filter tips (Axygen). Next, the cells were counted and assessed for viability using Trypan blue staining with a haemocytometer. During the dissociation procedure, the cells were kept on ice whenever possible, and the entire procedure was completed in < 90 min (generally ~70 min) to avoid the dissociation-associated artifacts recently described.^17^ A positive signal for a dissociation signature^17^ that reflects dissociation-associated changes in gene expression was obtained in < 1% of the cells (Supplementary information, Fig. S2a, b).

### Droplet-based scRNA-seq

A chromium single-cell 3′-library was constructed using the Chromium Single-cell 3′-Library, Gel Bead & Multiplex Kit and Chip Kit (10× Genomics, Pleasanton, CA, USA) according to the manufacturer’s instructions. Cell suspensions were loaded onto a chromium single-cell chip along with reverse transcription (RT) master mix and single-cell 3′-gel beads, aiming for 2000–8000 single cells per reaction. The samples were processed using 10× Genomics V2 barcoding chemistry kits (Supplementary information, Table S2). Following cell lysis, first-strand cDNA synthesis and amplification were carried out according to the manufacturer’s instructions with cDNA amplification set for 12 cycles. Libraries were sequenced on an Illumina HiSeq X Ten system, and mapped to the human reference genome (build hg19) and EBV B95–8 reference genome (NC_007605.1) using CellRanger (10× Genomics).

### WES and data analysis

DNA was extracted with the DNeasy Tissue and Blood Kit (Qiagen, Venlo, The Netherlands). Fragments (200–250 base pairs (bp) in length) were generated from the genomic DNA sample by Covaris (Covaris, Woburn, MA, USA) and end-repaired by the addition of an extra A base to the 3′ end. Illumina adapters (Illumina, San Diego, CA, USA) were ligated to both ends of the resulting fragments and amplified by PCR. After each step, the Agilent SureSelect Human All Exon V6 Kit (Agilent Technologies, Santa Clara, CA, USA) was used for whole-exome capture according to the manufacturer’s protocol. The final library was evaluated in two ways: determination of the average molecule length using an Agilent 2100 bioanalyser instrument (Agilent DNA 1000 Reagents) and quantification of the library by qRT-PCR (TaqMan Probe; Thermo Fisher Scientific). The resulting libraries were then sequenced using a HiSeq X Ten platform (Illumina). Sequencing reads were discarded if they contained adapter reads, low-quality reads, too many nitrogen atoms (> 10%), or low-quality bases (> 50% bases with quality < 5). High quality paired-end reads were then subjected to gapped alignment to a UCSC human reference genome (hg19) using BWA-MEM (v0.7.15). Picard (v1.84; http://broadinstitute.github.io/picard/) was used to sort and mark duplicate reads caused by PCR amplification. Local realignment and base quality score recalibration of the BWA-aligned reads were then conducted using the Genome Analysis Toolkit (GATK; v3.4, http://www.broadinstitute.org/gatk).^63^

### Bulk RNA-seq and data analysis

Total RNA was isolated from tissue samples using TRIzol reagent (Life Technologies, Carlsbad, USA). For RNA-seq, cDNA libraries were generated using a TruSeq RNA Sample Preparation kit (Illumina) according to the manufacturer’s protocol. After quality control of the cDNA libraries was performed, paired-end sequencing was carried out via HiSeq X Ten at BGI (Shenzhen). Paired-end reads were then mapped to the UCSC hg19 human transcriptome using Bowtie2. The expression levels of genes were quantified as fragments per kilobase of exon model per million mapped fragments (FPKM) calculated using RSEM (version 3.0.0) in the paired-end mode.

### Single-cell gene expression quantification

We used CellRanger (version 1.3.1) to generate a raw gene expression matrix for each scRNA-seq sample and used Single-Cell Remover of Doublets (Scrublet) (see below) to infer and remove cell doublets in each sample individually. Then the gene expression matrices for all tumor and normal samples were combined in R (version 3.4.1) and converted to a Seurat object using the Seurat R package (version 3.0.2).^15^ Quality filtering was performed to remove cells with < 201 or > 9000 expressed genes or > 20% unique molecular identifiers (UMIs) derived from the mitochondrial genome. In the remaining cells, gene expression matrices were log normalized to total cellular read-counts and mitochondrial read-counts by linear regression implemented using the ScaleData function of the Seurat package.

### Identification of the major cell types and their subtypes

We selected the 6000 most variably expressed genes to identify major cell types. To reduce dimensionality, principal component analysis was used to summarize the resulting variably expressed genes, and then the t-SNE dimensionality reduction (RunTSNE function) was used to further summarize the principal components. The number of principal components used was dataset dependent; they were estimated by an Elbow plot, in combination with exploration of the top genes from each principal component. The graph-based clustering approach implemented in the FindClusters function of the Seurat package was used for data clustering, with a *K* parameter of 30 and default parameters used otherwise. In brief, this method applies a shared nearest neighbor (SNN) modularity optimization-based clustering algorithm to identify cell clusters.^15,16^

We then annotated the clusters by the average expression of curated gene sets of the following major cell types: epithelial cells (*EPCAM*, *KRT19*, *KRT18*, *KRT5*, and *KRT15*), myeloid cells (*LYZ*, *CD68*, *MS4A6A*, *CD1E*, *IL3RA*, and *LAMP3*), T cells (*CD2* and *CD3D*/*E/G*), B cells (*CD79A*/*B*, *CD19*, and *MS4A1*), and CAFs (*COL1A1*, *COL3A1*, and *DCN*).

Then, we performed cluster-based doublet exclusion (see below). After doublet removal, we repeated the abovementioned steps (normalization, dimensionality reduction, and clustering) to identify the major cell types among the remaining cells. To identify subclusters within each major cell type, the cells belonging to each cell type were reanalyzed separately; we performed normalization, dimensionality reduction, and clustering as described above for each of the major cell types. Then, the subclusters were annotated to cell subtypes by the average expression of corresponding curated gene sets (Figs. 3b, 4b and 5b).

### Doublet removal

We applied Scrublet^64^ to computationally infer and remove doublets in each sample individually, with an expected doublet rate of 0.06 and default parameters used otherwise. The doublet score threshold was set by visual inspection of the histogram in combination with automatic detection. In addition to the analysis with Scrublet, we performed cluster-based doublet removal^65,66^; 13% of cells were excluded after doublet removal. Following the initial clustering, we performed clustering separately for each major cell type (i.e., epithelial, myeloid, T and B cells), removed the subclusters enriched in potential doublets, and then repeated the clustering of each major cell type. Thus, this was an iterative process alternating between removing doublets and clustering. Doublets were identified through annotation of subclusters using curated gene sets for the major cell types as described above.

### Comparison with bulk RNA-seq data

Comparisons of bulk RNA-seq data were performed with the 12 tumor samples with available bulk RNA-seq data. To compare the consistency of the bulk RNA-seq data with that of the scRNA-seq data, we compared read-counts per gene of bulk RNA-seq data normalized to gene length and total read-count directly to the sum of UMIs per gene for the corresponding tumor sample. Density dot plots and Pearson’s correlation (*r*) of the expression were generated.

### CNV analysis and identification of malignant epithelial cells

To separate malignant and non-malignant epithelial cells, we calculated CNVs in epithelial cells based on our scRNA-seq data using inferCNV (version 0.1) as described previously.^11,20^ Epithelial cells were identified by removing cells annotated as doublets after the initial process of major cell-type subclustering. Our data were transformed into log_2_(TPM + 1), where transcripts per million (TPM) was calculated as the proportion of UMIs of a gene within each cell multiplied by 1,000,000. CNV scores were computed on a moving average window equal to 101. The CNV scores of the epithelial cells from the normal sample were also calculated as a CNV control. We normalized the CNV profiles by subtracting the average expression profiles of the normal sample from the entire CNV dataset. The scores were restricted to the range −1 to 1 by replacing all values > 1 with 1 and all values < −1 with −1; any score between −0.3 and 0.3 was set to 0. We then performed hierarchical clustering of the CNV profiles of epithelial cells from the normal sample and each of the 15 tumor samples separately^21^; the non-malignant cells of the tumor samples were identified as those in the cluster that predominantly contained cells from the normal sample, while the cells with deletions and amplifications of entire chromosomes in the other cluster were identified as malignant cells.

To ensure that the CNV profiles we inferred using this method matched those that would have been found by direct measurement of DNA by WES, we additionally isolated DNA from bulk tumor tissue samples from each of the patients and generated DNA libraries directly.

### Identification of gene expression signatures reflecting the intratumoral transcriptional heterogeneity of malignant cells

Focusing on 7581 malignant cells from the 11 tumors from which the largest numbers of malignant cell transcriptomes were acquired, NMF was used to identify variably expressed metagenes. Unsupervised NMF was performed using the NMF R package (version 0.20.6).^67^ We selected *k* = 4 as the number of factors because it yielded a high cophenetic correlation coefficient^68^ and effectively decomposed the dataset of each tumor. A total of 44 metagenes were identified across the 11 tumors, and we listed the top-ranked genes according to their loadings of the NMF factor, as shown in Supplementary information, Table S4. The 44 metagenes were then compared by hierarchical clustering, using one minus the Pearson correlation coefficient over all gene scores as a distance metric. Five clusters of signatures were identified manually (Fig. 2c; Supplementary information, Table S5). For each signature, we then combined the top 100 genes of each metagene and calculated the average loadings for each gene. We summarized the total loadings for repetitive genes, retained the original loadings for exclusive genes, and divided the loadings of each gene by the number of metagenes within the signature. Finally, the top 30 genes with the highest loading were defined as the marker genes for the signature.

### Identification of the marker gene signatures of immune cell subclusters

To identify a marker gene signature for each of the 23 immune subclusters within the three cell types (i.e., myeloid, T and B cells), the FindMarkers function of Seurat was used to compare cells within the studied subcluster with all other cells of this cell type. Marker genes of subcluster were defined as having an average expression > 2.5-fold higher in the studied subcluster than in the other subclusters containing the same cell type, with detectable expression in > 25% of the cells in that subcluster. In addition, marker genes were required to have the highest mean expression in the studied subcluster compared to all others containing the same cell type. These parameters yielded marker genes for 17 subclusters (Supplementary information, Table S3); we failed to identify marker genes for 6 subclusters. The marker genes of each subcluster form the corresponding gene expression signature.

### Correlation and survival analyses with the gene expression signatures

We assessed the associations of the malignant and immune signatures with clinicopathological features and survival in two independent NPC cohorts, NPC Cohorts A (*n* = 113) and B (*n* = 128). NPC Cohort A, which was described by Zhang and colleagues^10^ (GSE102349), consisted of 113 NPC samples profiled by RNA-seq. The RNA-seq data were represented as FPKM, log_2_-transformed, and filtered to include only genes expressed in at least 50% of the samples. In addition, we retrospectively collected 128 stage II–IV, non-metastatic, pretreatment, formalin-fixed, paraffin-embedded (FFPE) samples between February 2010 and December 2015 at Sun Yat-sen University Cancer Center (NPC Cohort B) (Supplementary information, Table S8). Total RNA was extracted using a QIAGEN FFPE RNeasy kit (QIAGEN GmbH, Germany) and hybridized to an Affymetrix HTA 2.0 microarray according to the manufacturer’s instructions. The experimental procedures were described in our previous study.^69^ Disease-free survival was used as the endpoint and was available for 88 and 128 patients in NPC Cohorts A and B, respectively. Additionally, we included the TCGA HNSCC cohort to assess the prognostic values of these malignant and immune signatures derived from NPC in other head and neck cancers; no NPC samples were included in the HNSCC data from TCGA.^25^ The TCGA HNSCC cohort comprised 366 HNSCC samples with complete data for age, sex, smoking history, disease stage, and disease-free survival; level 3 data were obtained from the TCGA data portal (https://tcga-data.nci.nih.gov/tcga/). Gene expression values were represented as RNA-Seq by Expectation Maximization (RSEM; normalized within each sample to the upper quartile of total reads); only genes expressed in at least 50% of the samples were retained for analyses.

For correlation and survival analyses, the expression of each gene signature was evaluated using single-sample gene set enrichment analysis (ssGSEA; GenePattern module^70^). The ssGSEA scores were subsequently correlated with clinicopathological features (age, sex, smoking history, EBV infection status, T (tumor) and N (nodal) categories, and clinical stage) using the Wilcoxon rank-sum test and correlated with intratumoral and stromal TILs and mutational burden using the Spearman correlation test. To assess the prognostic values of the gene expression signatures, patients in cohorts were allocated into high and low expression groups according to the median value of the ssGSEA score for each signature gene set. Kaplan–Meier survival curves were plotted with the R Survival package to show differences in survival time. Hazard ratios (HRs) and adjusted *P* values were derived using a Cox proportional hazards model implemented in the R Survival package that included age, sex, smoking history (yes vs no) and tumor stage (III–IV vs I–II) in addition to the high and low expression groups of each signature; the pretreatment EBV DNA copy number (> 4000 vs ≤ 4000 copies/mL) was also included for NPC Cohort B.

### Trajectory analysis

Trajectory analysis was performed separately for the CD8^+^ T cells, CD4^+^ T_conv_ cells, and B cells using Monocle 2 (version 2.6.4).^71^ We then conducted differential gene expression analysis of the studied cells using the differentialGeneTest function to identify significant genes (BH-corrected *P* < 0.01), and cell ordering was performed on these genes in an unsupervised fashion. Trajectory construction was then performed after dimensionality reduction and cell ordering with default parameters. The gene signatures^31,72^ used to annotate Monocle components are listed in Supplementary information, Table S9.

### Gene set variation analysis

Predominantly, pathway analyses were carried out to evaluate activation of hallmark pathways and metabolic pathways, which were described in the molecular signature database^73^ and the curated dataset,^74^ respectively. Then, we applied Gene set variation analysis (GSVA)^75^ in the GSVA package (version 1.26.0) to assign pathway activity estimates to individual cells.

### SCENIC analysis

SCENIC analysis was conducted as described previously.^35^ We used the pySCENIC package (version 0.9.9), a lightning-fast python implementation of the SCENIC pipeline. Two gene-motif rankings (10 kb around the transcription start site (TSS) or 500 bp upstream of the TSS) were used to determine the search space around the TSS, and the 20-thousand motif database was used for RcisTarget and GENIE3.

### Cell–cell communication analysis

To investigate potential interactions across different cell types in the NPC TME, cell–cell communication analysis was performed using CellPhoneDB, which is a publicly available repository of curated receptors and ligands and their interactions.^50^ CellPhoneDB analysis was performed using the CellPhoneDB Python package (1.1.0).^50^ Single-cell transcriptomic data of cells annotated as tumor cells, macrophages, DC1, DC2, DC3, pDCs, monocytes, CD4^+^ T_conv_ cells, CD4^+^ Tregs, CD8^+^ T cells, CD8^+^ T_dysfunctional_, NK cells, B cells, GC B cells, and plasma cells were input into CellPhoneDB for cell–cell interaction analysis. Enriched receptor–ligand interactions between two cell types were derived based on the expression of a receptor by one cell type and the expression of the corresponding ligand by another cell type. Then, we identified the most relevant cell type-specific interactions between ligands and receptors, and only receptors and ligands expressed in more than 10% of the cells in the corresponding subclusters were considered.

Pairwise comparisons were performed between the included cell types. We first randomly permuted the cluster labels of all cells 1000 times to determine the mean of the average receptor and ligand expression levels of the interacting clusters. This generated a null distribution for each receptor–ligand pair. By calculating the proportion of the means that were higher than the actual mean, a *P* value for the likelihood of the cell type specificity of the corresponding receptor–ligand complex was obtained. We then selected interactions that were biologically relevant.

### Immunofluorescence analysis

Multiplex staining was performed using the PANO 7-plex IHC kit (Panovue, Beijing, China) according to the manufacturer’s instructions. Briefly, sections (4-μm thickness) obtained from paraffin-embedded samples were dewaxed, rehydrated, and subjected to high-temperature antigen retrieval. The tissues were incubated with blocking antibody diluent at room temperature for 10 min, and then incubated overnight at 4 °C with the following primary antibodies: anti-ACTA2 (mouse, A5228, dilution 1:10,000; Sigma), anti-CD31 (mouse, ZM-0044, dilution 1:300; OriGene), anti-EPCAM (rabbit, 14452S, dilution 1:150; Cell Signaling Technology), anti-CD4 (mouse, ZM-0418, dilution 1:100; OriGene), anti-CD8 (rabbit, ZA-0508, dilution 1:200; OriGene), anti-CD20 (rabbit, HPA014341, dilution 1:3000; Sigma), anti-IL3RA (mouse, ZM-0423, dilution 1:50; OriGene), anti-CD57 (mouse, ZM-0058, dilution 1:100; OriGene), anti-LAG-3 (rabbit, ab180187 dilution 1:500; Abcam), and anti-HAVCR2 (rabbit, 45208S, dilution 1:1000; Cell Signaling Technology). The slides were then incubated with the secondary antibody (HRP polymer, anti-mouse/Rabbit IgG) at room temperature for 10 min. Subsequently, fluorophore (tyramide signal amplification, TSA plus working solution) was applied to the tissues. The slides were microwave heat-treated after each TSA operation treatment and the primary antibodies were applied sequentially, followed by incubation with the secondary antibody and TSA treatment. Nuclei were stained with 4′-6′-diamidino-2-phenylindole (DAPI, Sigma-Aldrich) after all the antigens had been labeled. To obtain multispectral images, five randomly selected fields were scanned for each of the stained slides using the Mantra System (PerkinElmer, Waltham, MA, USA) at 200× magnification. The spectrum of autofluorescence of tissues and each fluorescein signal were extracted using images of unstained and single-stained sections. The extracted images were then used to establish a spectral library using inForm image analysis software (PerkinElmer), and reconstructed images of sections with the autofluorescence removed were obtained. The area of the nuclei was defined by detecting circular signals in the DAPI channel, and the percentages of certain cell subtypes were evaluated by dividing the colocalized signals for DAPI and the corresponding cell subtype marker by the DAPI-positive signal for each sample. Within the tumors, the area of tumor epithelial nests was determined morphologically by detecting aggregates of EPCAM^+^ cells.

For immunofluorescence confirmation of DC1 and DC3, the 4-μm paraffin-embedded sections were deparaffinized, rehydrated, subjected to blockade of endogenous peroxidase activity and high-temperature antigen retrieval, permeabilized in PBS with 0.5% Triton X-100, and incubated overnight at 4 °C with the following primary detection antibodies: anti-CD11c (mouse, ab11029, dilution 1:150; Abcam), anti-CLEC9A (rabbit, ab222794, dilution 1:100; Abcam), and anti-CCR7 (rabbit, ab32527, dilution 1:100; Abcam). The sections were incubated with Alexa Fluor 488-conjugated rabbit IgG and Alexa Fluor 594-conjugated mouse IgG secondary antibodies (1:1000; Life Technologies, Carlsbad, CA, USA; A-11008 or A-11001). Nuclei were counterstained with Hoechst 33342 (H3570, 2 μg/mL, Invitrogen, Grand Island, NY, USA). Images were captured using a confocal laser-scanning microscope (Olympus FV1000, Japan).

### Isolation of mononuclear cells from blood and tissues

Peripheral mononuclear cells were isolated from the peripheral blood of healthy donors collected at the Guangzhou Blood Center by Ficoll density gradient centrifugation. Specifically, monocytes were purified by a CD14^+^ cell isolation kit (Miltenyi Biotec), and left untreated or transfected with 300 nM negative control (NC) siRNA, or NR1H3/TFEC-specific siRNAs (EA-100) using P3 primary cell 4D-Nucleofector X kit (V4XP-3024, Lonza). Macrophages were obtained by exposing the monocytes to M-CSF (40 ng/mL, R&D) for 6 days with DMEM medium supplemented with 10% serum (Thermo Fisher Scientific). The CD8^+^ T cells and NK cells were purified by a CD8^+^ T cell isolation kit and a NK cell isolation kit, respectively (Miltenyi Biotec), and then left untreated or transfected with 300 nM NC siRNA, or Eomes/Runx3/XBP1-specific siRNAs (FI-115) using P3 primary cell 4D-Nucleofector X kit (V4XP-3024, Lonza). CD8^+^ T cells (2 × 10^5^ cells/well in 96-well plates) were activated by incubation with RPMI medium supplemented with 10% serum (Thermo Fisher Scientific) in the presence of 10 U/mL IL-2 (R&D) and 2.5 μg/mL anti-CD3 (mouse, 16-0038-85, 2.5 μg/μL; eBioscience) and anti-CD28 (mouse, 16-0089-85, 2.5 μg/μL; eBioscience) for 36 h. 2 × 10^5^ NK cells/well in 96-well plates were incubated in RPMI medium supplemented with 10% serum (Thermo Fisher Scientific) and 50 IU/mL IL-2 (R&D) for 16 h.

The NPC samples were washed with PBS (Thermo Fisher Scientific, Waltham, MA, USA), cut into small pieces (< 1 mm^3^) and transferred to 1 mL PBS containing collagenase IV (1 mg/mL) (Thermo Fisher Scientific) and 2% FBS. The samples were incubated for 30 min at 37 °C with manual shaking every 10 min and then filtered twice using a 40-µm nylon mesh (Thermo Fisher Scientific), and the mononuclear cells were washed and resuspended in RPMI 1640 supplemented with 10% FBS.

### Flow cytometry

The mononuclear leukocytes were labeled with monoclonal antibodies for 30 min on ice in the dark and washed with PBS containing 2% BSA (Sigma-Aldrich). The cells were washed, fixed, and permeabilized using the FOXP3/Transcription Factor Staining Buffer Set (eBioscience) according to the manufacturer’s instructions.

For analysis of primary human monocytes stimulated by M-CSF, cells were stained with anti-CD14 (mouse, 557923, dilution 1:120; BD Bioscience), anti-CD86 (mouse, 555660, dilution 1:100; BD Bioscience), and anti-HLA-DR (mouse, 562804, dilution 1:120; BD Bioscience) antibodies.

For analysis of CD8^+^ T cells and NK cells, cells were stained with anti-CD3 (mouse, 300405, dilution 1:100; BioLegend), anti-CD4 (mouse, 317418, dilution 1:100; BioLegend), anti-CD8 (mouse, 344708, dilution 1:100; BioLegend), and anti-CD56 (mouse, 318318, dilution 1:100; BioLegend) antibodies; CD8^+^ T cells and NK cells were gated as CD3^+^CD4^–^CD8^+^ cells and CD56^+^CD3^–^ cells, respectively. CD8^+^ T cells and NK cells were also stained with anti-GZMB (mouse, 372204, dilution 1:100; BioLegend), anti-perforin (mouse, 308122, dilution 1:100; BioLegend), anti-EOMES (mouse, 12-4877-41, dilution 1:100; eBioscience), PE-conjugated anti-RUNX3 (mouse, ab224641, dilution 1:500; Abcam) and anti-XBP1 (mouse, ab109221, dilution 1:80; Abcam) antibodies.

For analysis of B cells, cells were stained with anti-CD19 (mouse, 302206, dilution 1:100; BioLegend), anti-CD138 (mouse, 356527, dilution 1:100; BioLegend), and anti-CXCR5 (mouse, 356923, dilution 1:100; BioLegend) antibodies. GC B cells were gated as CD19^+^CD138^–^CXCR5^+^ cells, and the other B cells were gated as CD19^+^CD138^+^ or CD19^+^CD138^–^CXCR5^–^ cells. Plasma cells were gated as CD19^+^CD138^+^ cells, and the other B cells were gated as CD19^+^CD138^–^ cells. B cells were also stained with anti-BCL6 (mouse, NBP2-59597, dilution 1:100; Novus Biologicals) and PE-conjugated anti-ATF4 (mouse, ab184909, dilution 1:80; Abcam) antibodies. Appropriate PE-conjugated isotype controls (rabbit, ab172730, diluted to the same concentration of the primary antibody; Abcam) were used for setting gates. Flow cytometry analyses were performed on a CytoFLEX flow cytometer (Beckman Coulter, Inc.) with FlowJo software (Tree Star Inc., Ashland, OR, USA). The gating strategy was shown in Supplementary information, Fig. S12.

### Immunoblotting

Proteins from cells were extracted as previously described.^76^ The following antibodies were used in immunoblotting: anti-NR1H3 (Rabbit, ab176323, dilution 1:1000; Abcam), anti-TFEC (Rabbit, ab185226, dilution 1:1000; Abcam), anti-EOMES (Rabbit, 81493s, dilution 1:1000; CST), anti-RUNX3 (Rabbit, ab224641, dilution 1:1000; Abcam), anti-XBP1 (Rabbit, ab109221, dilution 1:1000; Abcam), and anti-β-actin (mouse, 3700s, dilution 1:1000; CST).

### Real-time PCR

TRIzol reagent (Invitrogen) was used to isolate total RNA from monocytes and macrophages as described above. The concentration and quality of the total RNA were determined using NanoDrop 2000. First-strand complementary DNA was synthesized using random primers and the M-MLV reverse transcriptase (Promega). PCR was performed in triplicate using SYBR Green qPCR SuperMix-UDG reagents (Invitrogen) on a CFX96 Touch sequence detection system (Bio-Rad). *GAPDH* was used as an endogenous control for all the genes. The primer sequences are listed in Supplementary information, Table S10.

### Statistical analysis

All statistical analyses were performed using R (http://www.r-project.org) and SPSS version 19.0 (SPSS Inc., Chicago, IL, USA). We used the R Base package with default parameters to generate box plots. The Beanplot R package was used to generate violin plots, with the data distribution bandwidth evaluated by kernel density estimation. We do not display each data point in all box and violin plots because the large number of data points would obscure the overall distribution. A two-sided paired or unpaired Student’s *t*-test and unpaired Wilcoxon rank-sum test was used where indicated. *P* < 0.05 was considered to indicate statistical significance.

## Supplementary information

Supplementary information, Fig. S1

Supplementary information, Fig. S2

Supplementary information, Fig. S3

Supplementary information, Fig. S4

Supplementary information, Fig. S5

Supplementary information, Fig. S6

Supplementary information, Fig. S7

Supplementary information, Fig. S8

Supplementary information, Fig. S9

Supplementary information, Fig. S10

Supplementary information, Fig. S11

Supplementary information, Fig. S12

Supplementary information, Table S1

Supplementary information, Table S2

Supplementary information, Table S3

Supplementary information, Table S4

Supplementary information, Table S5

Supplementary information, Table S6

Supplementary information, Table S7

Supplementary information, Table S8

Supplementary information, Table S9

Supplementary information, Table S10

## Data Availability

Raw scRNA-seq data were deposited in the CNGB Nucleotide Sequence Archive (accession code: CNP0000428) and the processed gene expression data can be accessed from Gene Expression Omnibus (accession code: GSE150430). The single-cell data from this study can be analyzed and visualized via a website portal (db.cngb.org/npcatlas). All other relevant data are available upon request. The authenticity of this manuscript has been validated by uploading the key raw data to the Research Data Deposit public platform (www.researchdata.org.cn) under the approval RDD number RDDB2019000024.

## Code availability

R and other custom scripts for analyzing data are available upon request.
