# Supplementary material for: Single-cell transcriptomics reveals regulators underlying immune cell diversity and immune subtypes associated with prognosis in nasopharyngeal carcinoma
Source: Cell Res. 2020 Jul 20;30(11):1024–42. doi: 10.1038/s41422-020-0374-x (PMC7784929; doi:10.1038/s41422-020-0374-x)
Supplement: Supplementary file 1 — Supplementary information, Fig. S1 [file 41422_2020_374_MOESM1_ESM.pdf]

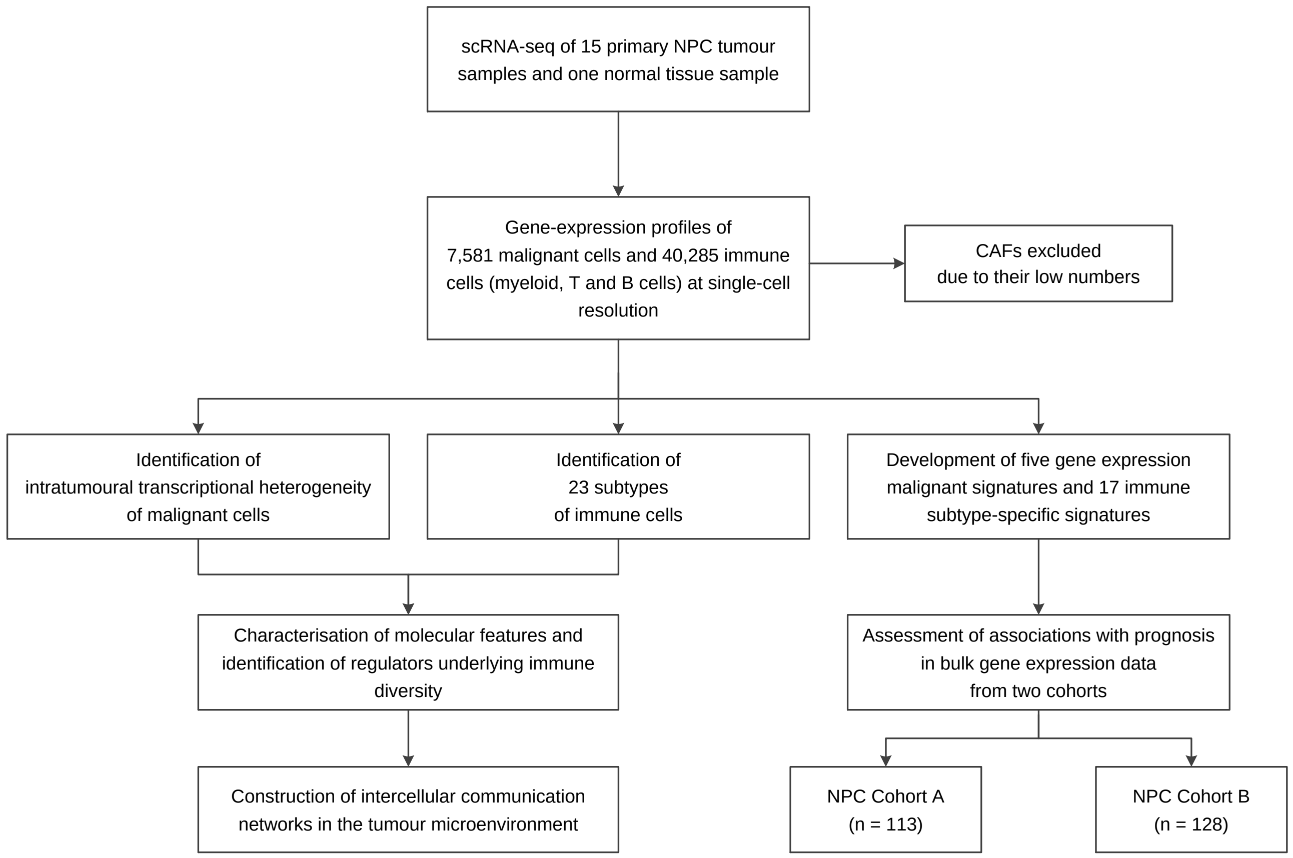

Fig. S1. Study flow chart. NPC, nasopharyngeal carcinoma; scRNA-seq, single-cell RNA sequencing; and CAF, cancer-associated fibroblast.
