# Supplementary material for: Single-cell transcriptomics reveals regulators underlying immune cell diversity and immune subtypes associated with prognosis in nasopharyngeal carcinoma
Source: Cell Res. 2020 Jul 20;30(11):1024–42. doi: 10.1038/s41422-020-0374-x (PMC7784929; doi:10.1038/s41422-020-0374-x)
Supplement: Supplementary file 2 — Supplementary information, Fig. S2 [file 41422_2020_374_MOESM2_ESM.pdf]

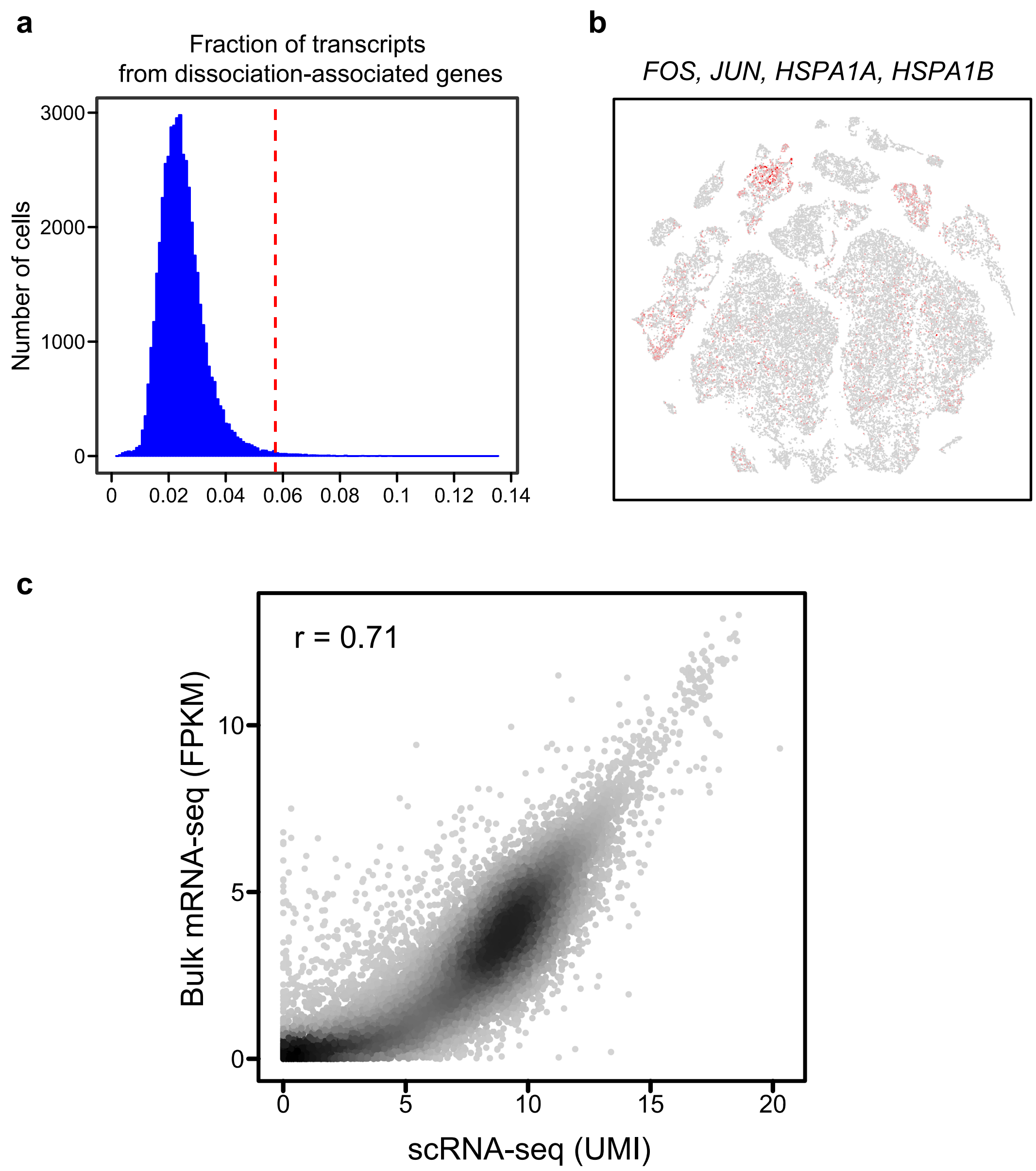

Fig. S2. Effects of cell dissociation and the correlation between bulk RNA-seq and scRNA-seq data. a, Histogram showing the fraction of gene expression attributed to dissociation-associated genes in the 48,584 cells profiled in this study. The red dotted line corresponds to the threshold described by Van Oudenaerden *et al.*[17] Their signature is based on detecting the upregulation of the expression of immediate-early genes (*FOS* and *JUN*) and stress-response genes (e.g., *HSPA1A* and *HSPA1B*). The plot shows that only a few cells (0.60%) are positive for the dissociation-response signature. Dissociation-associated artefacts were thus unlikely to be a major determinant of the cell subtypes identified in this study. **b,** tSNE plot of 48,584 cells colour coded for expression of the gene set (*FOS, JUN, HSPA1A,* and *HSPA1B*). **c,** Density dot plot and Pearson's correlation analysis (r) of the gene expression in the scRNA-seq and bulk mRNA-seq data of 12 NPC samples.
