# Supplementary material for: Single-cell transcriptomics reveals regulators underlying immune cell diversity and immune subtypes associated with prognosis in nasopharyngeal carcinoma
Source: Cell Res. 2020 Jul 20;30(11):1024–42. doi: 10.1038/s41422-020-0374-x (PMC7784929; doi:10.1038/s41422-020-0374-x)
Supplement: Supplementary file 3 — Supplementary information, Fig. S3 [file 41422_2020_374_MOESM3_ESM.pdf]

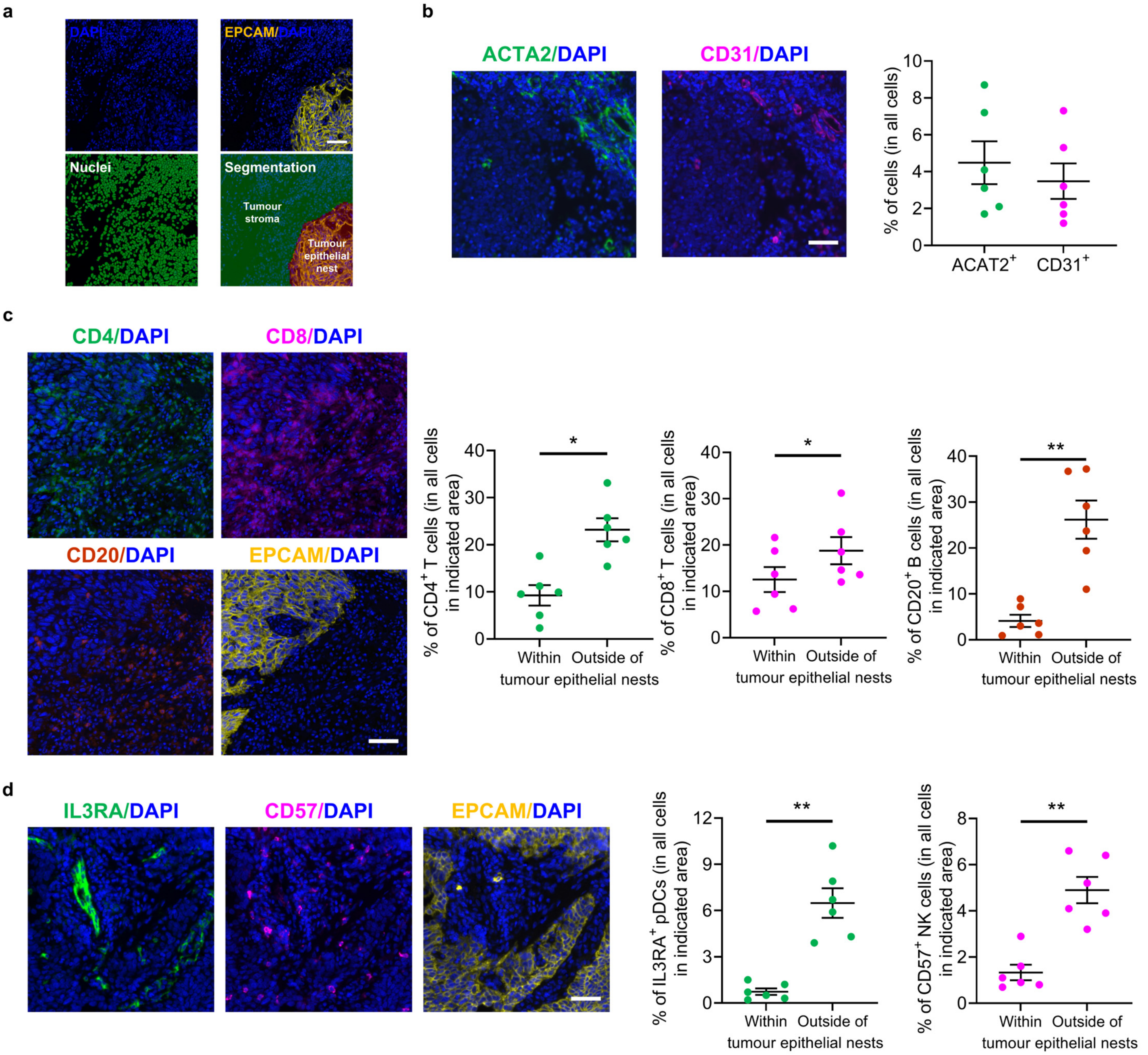

Fig. S3. Multiplex immunofluorescence staining and automated image analysis. a, Schematic illustration of the automated image analysis of multiplex immunofluorescence staining. **b**, Left, representative multiplex immunofluorescence images for ACTA2 and CD31 in an NPC tumour sample. Right, quantitation of ACTA2$^+$ and CD31$^+$ cells as a percentage of all cells (n = 6). mean ± s.e.m. **c**, Left, representative multiplex immunofluorescence images for CD4, CD8, CD20 and EPCAM in an NPC tumour sample. Right, quantitation of the percentages of CD4$^+$, CD8$^+$, and CD20$^+$ cells within and outside of the area of tumour epithelial nests (n = 6). mean ± s.e.m. **d**, Left, representative multiplex immunofluorescence images for IL3RA, CD57 and EPCAM in an NPC tumour sample. Right, quantitation of the percentage of IL3RA$^+$ and CD57$^+$ cells within and outside of the area of tumour epithelial nests (n = 6). mean ± s.e.m. Scale bar: 50 μm. $^*P$ < 0.05, and $^{**}P$ < 0.01 (paired Student's t-test).
