# Supplementary material for: Single-cell transcriptomics reveals regulators underlying immune cell diversity and immune subtypes associated with prognosis in nasopharyngeal carcinoma
Source: Cell Res. 2020 Jul 20;30(11):1024–42. doi: 10.1038/s41422-020-0374-x (PMC7784929; doi:10.1038/s41422-020-0374-x)
Supplement: Supplementary file 4 — Supplementary information, Fig. S4 [file 41422_2020_374_MOESM4_ESM.pdf]

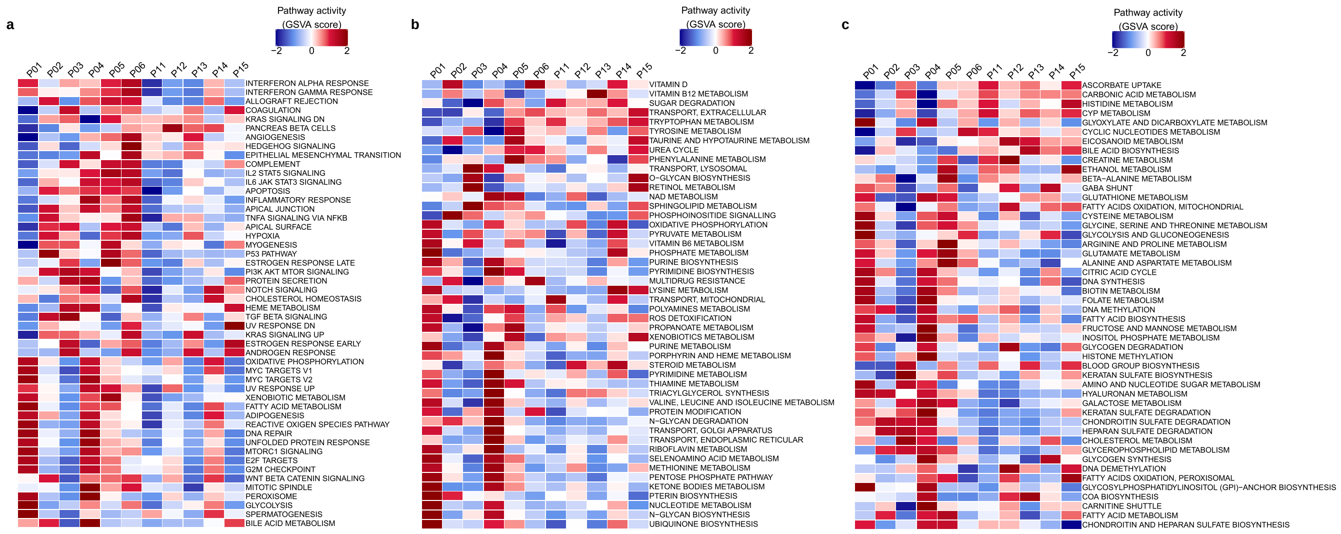

Fig. S3. Differences in pathway activities among malignant cells from different patients. a-c, Differences in the activities of the HALLMARK pathway (a) and metabolic pathways (b, c) in the malignant cells from 11 patients scored per cell by GSVA. The scores of pathways are normalized.
