# Supplementary material for: Single-cell transcriptomics reveals regulators underlying immune cell diversity and immune subtypes associated with prognosis in nasopharyngeal carcinoma
Source: Cell Res. 2020 Jul 20;30(11):1024–42. doi: 10.1038/s41422-020-0374-x (PMC7784929; doi:10.1038/s41422-020-0374-x)
Supplement: Supplementary file 5 — Supplementary information, Fig. S5 [file 41422_2020_374_MOESM5_ESM.pdf]

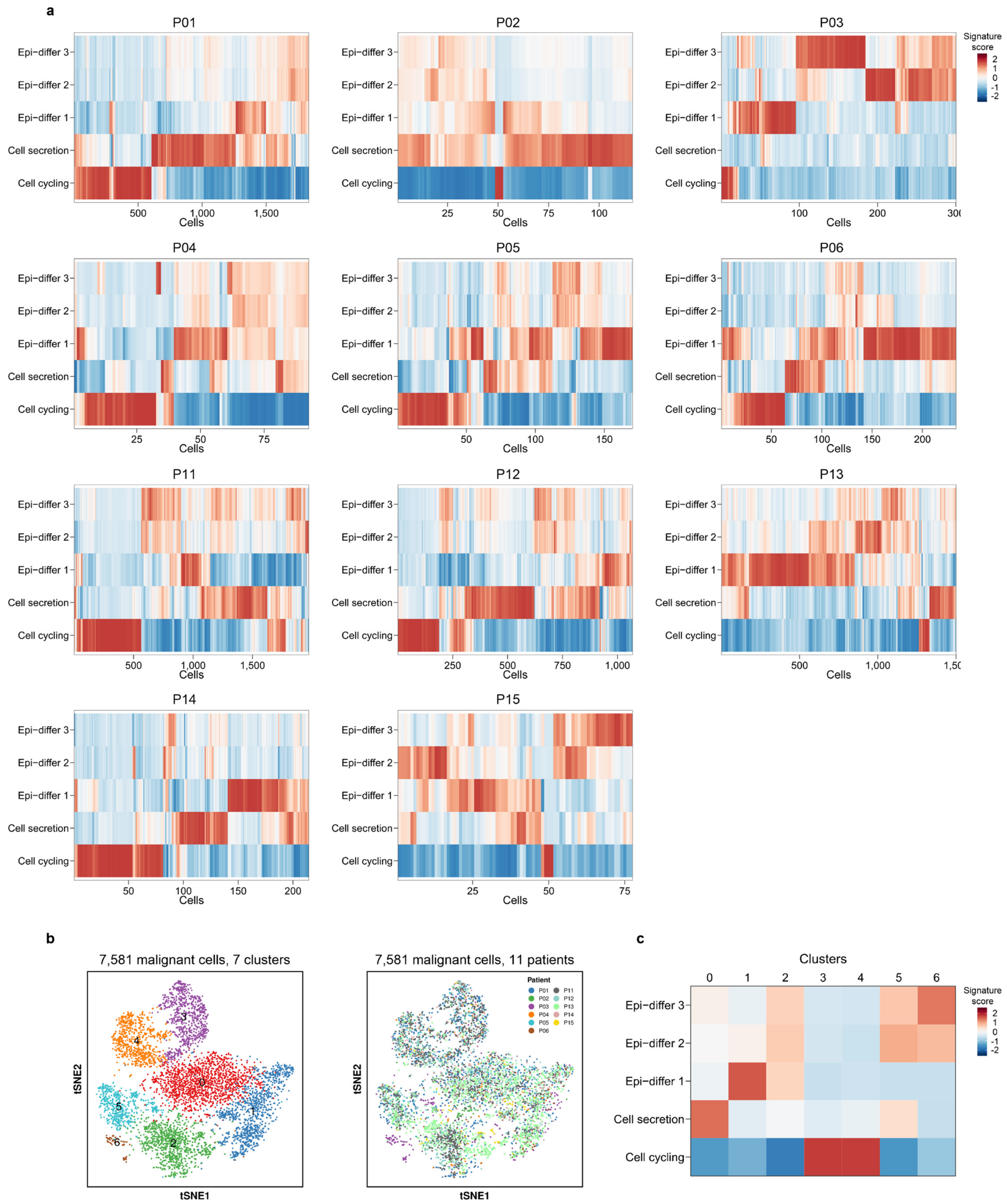

Fig. S5. **a**, Heatmaps showing the expression of five malignant signatures in malignant cells (columns) from 11 tumour samples containing at least 50 malignant cells. **b**, tSNE plot of 7,581 malignant cells from 11 patients clustered using only genes from the malignant signatures, with each cell colour-coded to indicate the associated cell clusters (left) and corresponding patients (right). **c**, Heatmap showing the average expression of five malignant signatures in each cell cluster shown in (b).
