# Supplementary material for: Single-cell transcriptomics reveals regulators underlying immune cell diversity and immune subtypes associated with prognosis in nasopharyngeal carcinoma
Source: Cell Res. 2020 Jul 20;30(11):1024–42. doi: 10.1038/s41422-020-0374-x (PMC7784929; doi:10.1038/s41422-020-0374-x)
Supplement: Supplementary file 6 — Supplementary information, Fig. S6 [file 41422_2020_374_MOESM6_ESM.pdf]

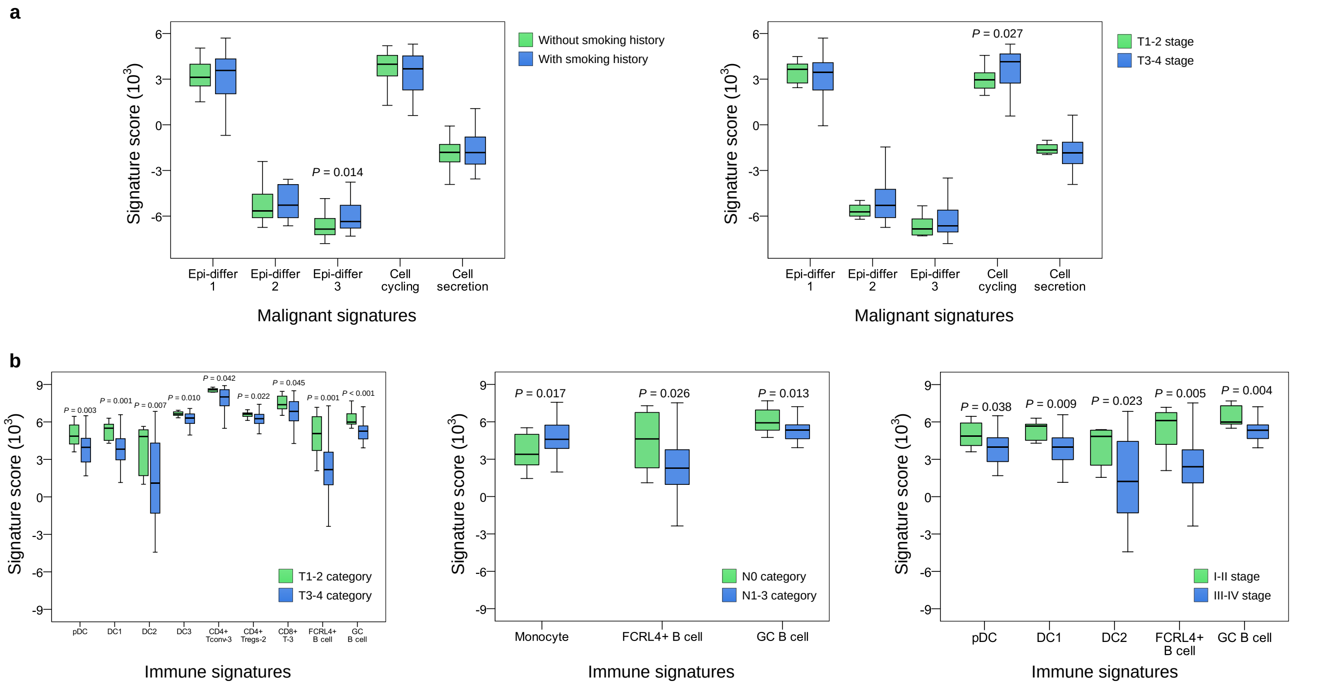

Fig. S6. Correlations of malignant and immune signatures with clinicopathological features in NPC. a, Changes in gene expression for the indicated five malignant signatures as a result of differences in smoking history and T category in NPC Cohort A. **b,** Changes in gene expression for the indicated immune signatures as a result of differences in T and N categories and clinical stage in NPC Cohort A. The box plot centre corresponds to the median, with the box and whiskers corresponding to the interquartile range and 1.5×interquartile range, respectively. *P*-values were based on the Wilcoxon rank-sum test.
