# Supplementary material for: Single-cell transcriptomics reveals regulators underlying immune cell diversity and immune subtypes associated with prognosis in nasopharyngeal carcinoma
Source: Cell Res. 2020 Jul 20;30(11):1024–42. doi: 10.1038/s41422-020-0374-x (PMC7784929; doi:10.1038/s41422-020-0374-x)
Supplement: Supplementary file 7 — Supplementary information, Fig. S7 [file 41422_2020_374_MOESM7_ESM.pdf]

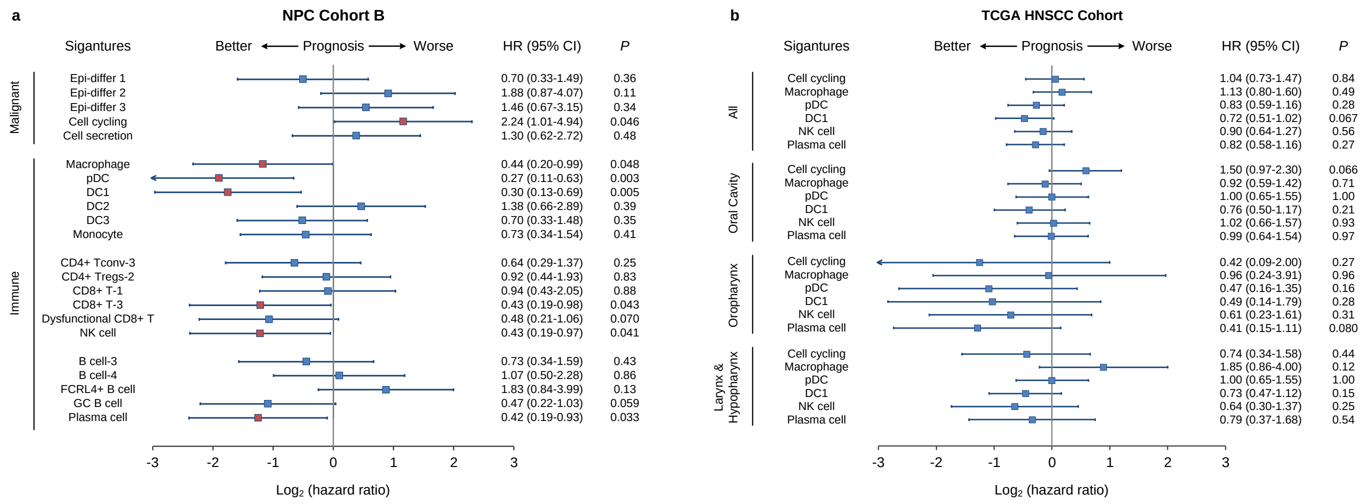

Fig. S7. Associations of gene signatures with survival in NPC and HNSCC. a, Prognostic values of the NPC signatures in 128 patients in NPC Cohort B. Forest plots show HRs (blue/red squares) and CIs (horizontal ranges) derived from multivariate Cox regression survival analyses for disease-free survival adjusted for age, sex, smoking history, disease stage, and pretreatment EBV DNA copy number in NPC; the corresponding Cox regression *P*-values are also shown. Significant results are indicated with red squares. **b,** Prognostic values of the HNSCC signatures in 366 patients in a TCGA HNSCC cohort (all samples and stratification by primary site). The corresponding prognostic signatures in NPC (i.e., cell cycling, macrophages, pDCs, *CLEC9A*$^{+}$ DC1, NK cells, and plasma cells) were obtained from two HNSCC scRNA-seq studies (Supplementary information, Table S7). As *CLEC9A*$^{+}$ DC1 was not identified in HNSCC studies, the NPC DC1 signature was used instead to infer its prognostic value in HNSCC. Forest plots show HRs (blue/red squares) and CIs (horizontal ranges) derived from multivariate Cox regression survival analyses for disease-free survival adjusted for age, sex, smoking history and disease stage; the corresponding Cox regression *P*-values are also shown. Significant results are indicated with red squares.
