# Supplementary material for: Single-cell transcriptomics reveals regulators underlying immune cell diversity and immune subtypes associated with prognosis in nasopharyngeal carcinoma
Source: Cell Res. 2020 Jul 20;30(11):1024–42. doi: 10.1038/s41422-020-0374-x (PMC7784929; doi:10.1038/s41422-020-0374-x)
Supplement: Supplementary file 8 — Supplementary information, Fig. S8 [file 41422_2020_374_MOESM8_ESM.pdf]

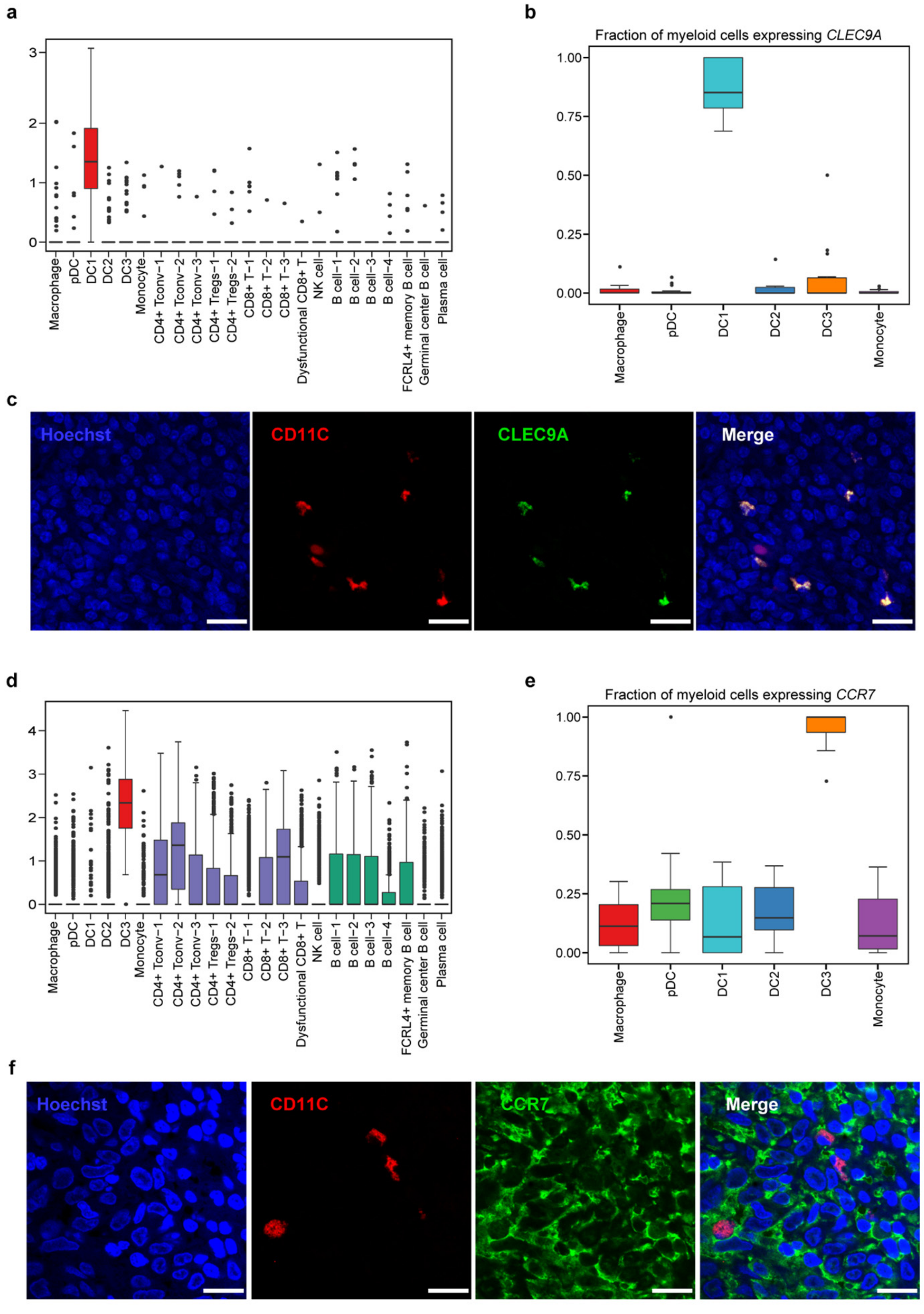

Fig. S8. Confirmation of the existence of CLEC9A$^+$ and CCR7$^+$ subsets of dendritic cells (DCs) in NPC. DCs express CD11c. **(a, d)** Box plots of *CLEC9A* (a) and *CCR7* (d) expression in the 23 immune cell subtypes, illustrating cell subtype-specific expression (DC1 and DC3 express *CLEC9A* and *CCR7*, respectively). The box plot centre corresponds to the median, with the box, whiskers and points corresponding to the interquartile range, 1.5×interquartile range, and outliers, respectively. **(b, e)** Box plot quantification of the fraction of each myeloid cell subtype expressing CLEC9A (b) or CCR7 (e). Box plot center corresponds to median, with box, whisker, and points corresponding to interquartile range, 1.5×interquartile range, and outliers respectively. **(c, f)** Immunofluorescence images of (c) CD11c and CLEC9A staining or (f) CD11c and CCR7 staining of an NPC tissue sample. Images are representative of seven independent samples for (c) and nine independent samples for (f). Scale bar: 20 μm.
