# Supplementary material for: Single-cell transcriptomics reveals regulators underlying immune cell diversity and immune subtypes associated with prognosis in nasopharyngeal carcinoma
Source: Cell Res. 2020 Jul 20;30(11):1024–42. doi: 10.1038/s41422-020-0374-x (PMC7784929; doi:10.1038/s41422-020-0374-x)
Supplement: Supplementary file 9 — Supplementary information, Fig. S9 [file 41422_2020_374_MOESM9_ESM.pdf]

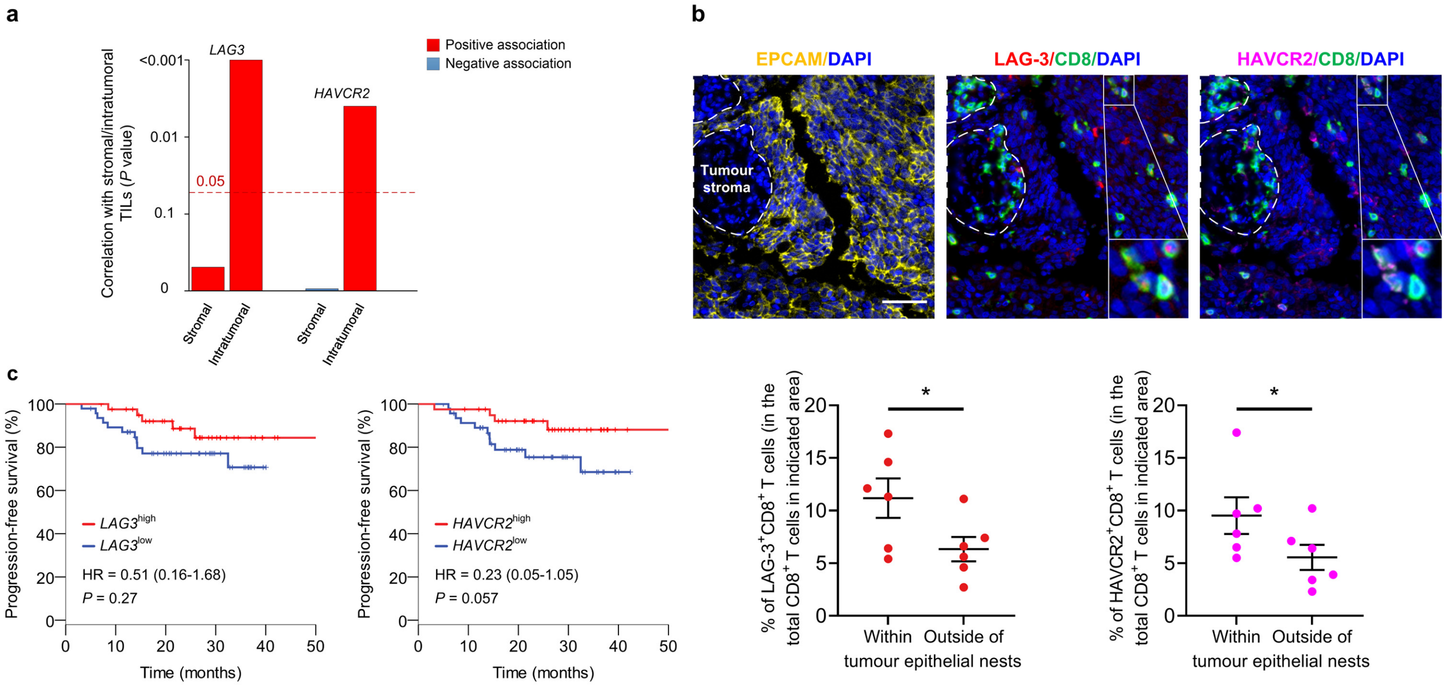

Fig. S9. **a**, Bar plot showing the direction and statistical significance (*P*-values were based on the Spearman correlation test) of the associations between stromal/intratumoural tumour-infiltrating lymphocytes (TILs) and the expression of *LAG3* and *HAVCR2* in the NPC Cohort A. **b**, Upper, representative multiplex immunofluorescence images for EPCAM, LAG-3, HAVCR2 and CD8 in an NPC tumour sample. The area within the dash line indicates the tumour stroma; the other area with aggregates of EPCAM$^+$ cells indicates the tumour epithelial nests. Lower, quantitation of LAG-3$^+$ and HAVCR2$^+$ CD8$^+$ T cells as a percentage of the total CD8$^+$ T cells within and outside of the area of tumour epithelial nests (n = 6). mean ± s.e.m. Scale bar: 50 μm. $^*P < 0.05$ (paired Student's *t*-test). **c**, Kaplan–Meier curves showing progression-free survival of the 88 patients in the NPC Cohort A stratified according to high versus low expression of *LAG3* and *HAVCR2*. Cox regression HRs and 95% CIs obtained after correcting for age, sex, smoking history and disease stage are shown; the corresponding Cox regression *P*-values are also shown.
