# Supplementary material for: Single-cell transcriptomics reveals regulators underlying immune cell diversity and immune subtypes associated with prognosis in nasopharyngeal carcinoma
Source: Cell Res. 2020 Jul 20;30(11):1024–42. doi: 10.1038/s41422-020-0374-x (PMC7784929; doi:10.1038/s41422-020-0374-x)
Supplement: Supplementary file 10 — Supplementary information, Fig. S10 [file 41422_2020_374_MOESM10_ESM.pdf]

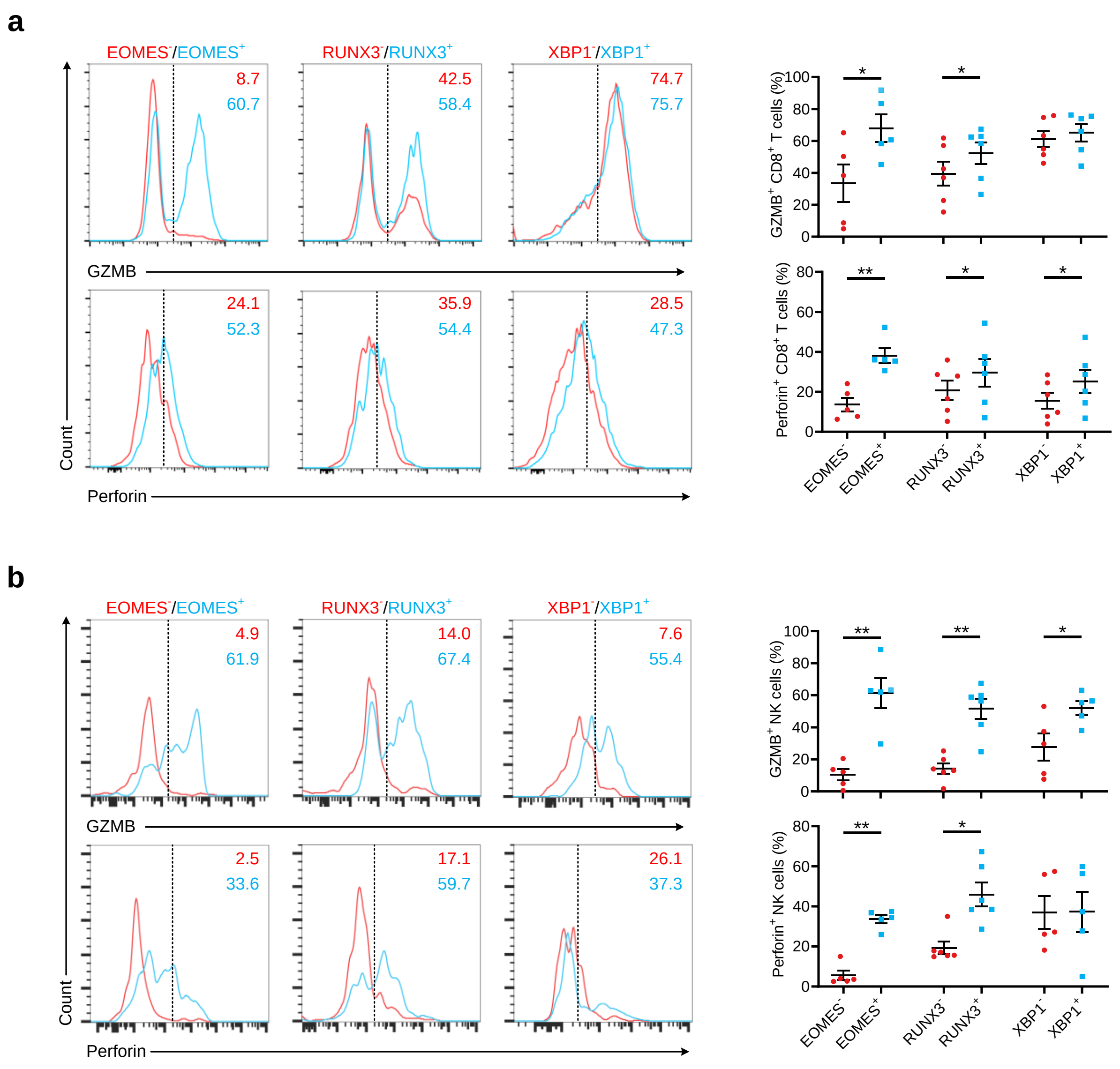

Fig. S10. Correlations between the expression of EOMES/RUNX3/XBP1 and GZMB/perforin in CD8$^+$ T cells and NK cells in NPC. a, Left, representative histograms depicting the expression of GZMB and perforin on EOMES$^-$ and EOMES$^+$ (*n* = 5 donors, *n* = 3 independent experiments), RUNX3$^-$ and RUNX3$^+$ (*n* = 6 donors, *n* = 6 independent experiments), and XBP1$^-$ and XBP1$^+$ (*n* = 6 donors, *n* = 5 independent experiments) CD8$^+$ T cells from tumour tissues. Right, associations of EOMES$^+$ (*n* = 5), RUNX3$^+$ (*n* = 6), and XBP1$^+$ (*n* = 6) CD8$^+$ T cells with GZMB$^+$ or perforin$^+$ CD8$^+$ T cells from tumour tissues. **b**, Left, representative histograms depicting the expression of GZMB and perforin on EOMES$^-$ and EOMES$^+$ (*n* = 5 donors, *n* = 4 independent experiments), RUNX3$^-$ and RUNX3$^+$ (*n* = 6 donors, *n* = 6 independent experiments), and XBP1$^-$ and XBP1$^+$ (*n* = 5 donors, *n* = 5 independent experiments) NK cells from tumour tissues. Right, associations of EOMES$^+$ (*n* = 5), RUNX3$^+$ (*n* = 6), and XBP1$^+$ (*n* = 5) NK cells with GZMB$^+$ or perforin$^+$ NK cells from tumour tissues. $^*P < 0.05$, and $^{**}P < 0.01$ (paired Student's *t*-test). Error bars, s.e.m.
