# Supplementary material for: Single-cell transcriptomics reveals regulators underlying immune cell diversity and immune subtypes associated with prognosis in nasopharyngeal carcinoma
Source: Cell Res. 2020 Jul 20;30(11):1024–42. doi: 10.1038/s41422-020-0374-x (PMC7784929; doi:10.1038/s41422-020-0374-x)
Supplement: Supplementary file 11 — Supplementary information, Fig. S11 [file 41422_2020_374_MOESM11_ESM.pdf]

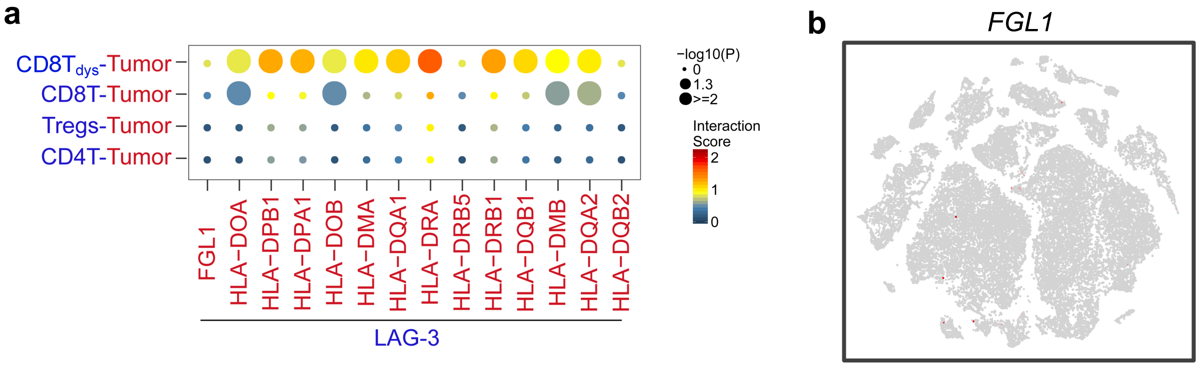

Fig. S11. Ligands-LAG-3 interactions between T cells and tumour cells in the NPC TME. a, Interactions of LAG-3 and its ligands, fibrinogen-like protein 1 (FGL1) and the major histocompatibility complex class II (MHC-II) protein complex, between four subtypes of T cells ($CD8T_{dys}$, CD8T, Tregs, and $CD4T_{conv}$) and tumour cells in the TME of NPC. *P*-values are indicated by circle size, with the scale to the right (permutation test). The means of the average expression levels of interacting molecule 1 in cluster 1 and interacting molecule 2 in cluster 2 are indicated by colour. Assays were carried out at the mRNA level but used to extrapolate protein interactions. **b,** tSNE plot of tumour cells, color coded for the expression of *FGL1*. $CD8T_{dys}$, dysfunctional CD8+ T cell; CD8T, CD8+ T cell; $CD4T_{conv}$, conventional CD4+ T cell.
