# Supplementary material for: Single-cell transcriptomics reveals regulators underlying immune cell diversity and immune subtypes associated with prognosis in nasopharyngeal carcinoma
Source: Cell Res. 2020 Jul 20;30(11):1024–42. doi: 10.1038/s41422-020-0374-x (PMC7784929; doi:10.1038/s41422-020-0374-x)
Supplement: Supplementary file 12 — Supplementary information, Fig. S12 [file 41422_2020_374_MOESM12_ESM.pdf]

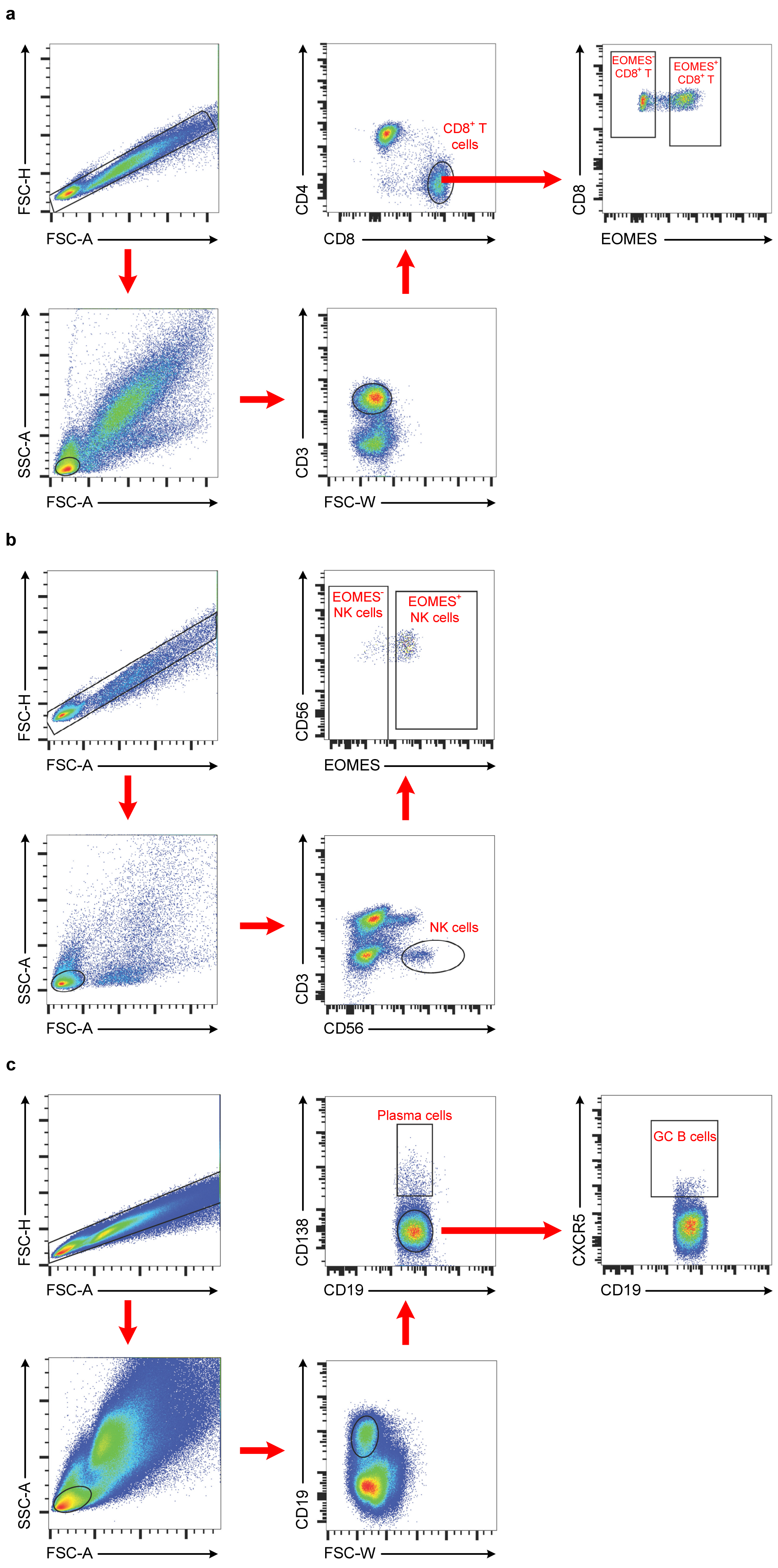

Fig. S12. Flow cytometry gating strategy for identification of tumor-infiltrating leukocytes in NPC. a, Identification of EOMES$^+$ and EOMES$^-$ CD8$^+$ T cells. **b,** Identification of EOMES$^+$ and EOMES$^-$ NK cells. **c,** Identification of plasma cells and GC B cells. Identified cell types are marked by red text.
