# Supplementary material for: Single-cell transcriptomics reveals regulators underlying immune cell diversity and immune subtypes associated with prognosis in nasopharyngeal carcinoma
Source: Cell Res. 2020 Jul 20;30(11):1024–42. doi: 10.1038/s41422-020-0374-x (PMC7784929; doi:10.1038/s41422-020-0374-x)
Supplement: Supplementary file 13 — Supplementary information, Table S1 [file 41422_2020_374_MOESM13_ESM.pdf]

Table S1. Characteristics of the 15 patients with NPC and one patient with chronic nasopharyngitis included in this study

Patient ID	Sex	Age	Histology	TNM stage*	Clinical stage*	Smoking history	Pretreatment plasma EBV DNA (copies/mL)	Bulk WES	Bulk RNA-seq	EBV status†
P01	Male	37	NKNPC	T3N1M0	III	Yes	$1.16 * 10^4$	Yes	Yes	+
P02	Female	38	NKNPC	T2N3M0	IVA	No	$1.91 * 10^3$	Yes	Yes	+
P03	Female	31	KNPC	T1N2M0	III	No	0	Yes	Yes	-
P04	Male	58	NKNPC	T4N3M1	IVB	Yes	0	Yes	Yes	+
P05	Male	74	NKNPC	T3N0M0	III	No	$1.38 * 10^3$	Yes	Yes	+
P06	Female	36	NKNPC	T2N2M0	III	No	$1.23 * 10^4$	Yes	Yes	+
P07	Male	45	NKNPC	T3N1M0	III	No	$7.90 * 10^2$	Yes	Yes	+
P08	Male	61	NKNPC	T2N1M0	II	Yes	$2.84 * 10^3$	Yes	Yes	+
P09	Male	51	NKNPC	T2N3M0	IVA	No	0	No	No	+
P10	Male	57	NKNPC	T4N1M0	IVA	Yes	0	Yes	Yes	-
P11	Male	44	NKNPC	T1N1M0	II	Yes	0	Yes	Yes	+
P12	Male	32	NKNPC	T4N2M0	IVA	No	$9.90 * 10^3$	Yes	Yes	+
P13	Female	41	NKNPC	rT2N0M0	rII	No	0	No	No	+
P14	Female	54	NKNPC	T4N2M1	IVB	No	$7.00 * 10^6$	Yes	Yes	+
P15	Male	32	NKNPC	T2N0M0	II	No	$1.06 * 10^2$	No	No	+
N01	Female	20	CNP	--	--	No	0	No	No	-

*According to the 8th American Joint Committee on Cancer (AJCC)/Union for International Cancer Control (UICC) staging system.

† Detected by In situ hydridization to the EBV-encoded small RNAs (EBERs).

NPC, nasopharyngeal carcinoma; NKNPC, non-keratinizing NPC; KNPC, keratinizing NPC; CNP, chronic nasopharyngitis; EBV, Epstein-Barr virus; WES, whole-exome sequencing.
