# Supplementary material for: Single-cell transcriptomics reveals regulators underlying immune cell diversity and immune subtypes associated with prognosis in nasopharyngeal carcinoma
Source: Cell Res. 2020 Jul 20;30(11):1024–42. doi: 10.1038/s41422-020-0374-x (PMC7784929; doi:10.1038/s41422-020-0374-x)
Supplement: Supplementary file 14 — Supplementary information, Table S2 [file 41422_2020_374_MOESM14_ESM.pdf]

Table S2. Sequencing statistics for the 15 NPC samples and one normal nasopharyngeal epithelial sample included in this study

Patient ID	Sample type	10X version*	UMIs	Cells	UMIs/cell
P01	Tumour	V2	112,899,534	2,217	50,924
P02	Tumour	V2	46,012,984	6,092	7,553
P03	Tumour	V2	57,160,298	6,135	9,317
P04	Tumour	V2	8,930,200	320	27,907
P05	Tumour	V2	24,831,459	1,842	13,481
P06	Tumour	V2	22,665,938	2,222	10,201
P07	Tumour	V2	10,146,149	1,931	5,254
P08	Tumour	V2	24,955,311	5,313	4,697
P09	Tumour	V2	12,251,927	834	14,691
P10	Tumour	V2	11,885,458	2,019	5,887
P11	Tumour	V2	40,914,978	2,589	15,803
P12	Tumour	V2	28,407,803	1,914	14,842
P13	Tumour	V2	31,000,178	2,524	12,282
P14	Tumour	V2	32,323,223	2,497	12,945
P15	Tumour	V2	44,970,146	7,552	5,955
N01	Normal	V2	23,341,939	2,583	9,037

*Single-cell suspensions were converted into single-cell RNA-seq libraries using a commercially available DROP-seq protocol (10X genomics) following version 2 chemistry.

NPC, nasopharyngeal carcinoma; UMI, unique molecular identifier, equivalent to a unique detected transcript.
