# Supplementary material for: Single-cell transcriptomics reveals regulators underlying immune cell diversity and immune subtypes associated with prognosis in nasopharyngeal carcinoma
Source: Cell Res. 2020 Jul 20;30(11):1024–42. doi: 10.1038/s41422-020-0374-x (PMC7784929; doi:10.1038/s41422-020-0374-x)
Supplement: Supplementary file 15 — Supplementary information, Table S3 [file 41422_2020_374_MOESM15_ESM.pdf]

Table S3. Immune cell cluster characteristics

Cluster	Annateted Name	Cells	Genes (Mean)	UMIs (Mean)	Marker Genes
Myeloid 0	Macrophage	1,837	2,840	12,269	APOE, APOC1, RNASE1, CTSL, C1QA, C1QB, C1QC, CTSD, ADAMDEC1, SPP1, FTL, NUPR1, GPNMB, FCGR3A, CCL8, PLD3, LGMN, GLUL, CTSC, CD163, PSAP, CTSB, CCL3, HES1, MMP9, MS4A4A, IFIT1, ACP5, PLA2G7, CD68, MAFB, CD14, IFI6, TREM2, MS4A7, PRDX1, ASAH1, LGALS3, CCL4, IFI27, MARCKS, CREG1, CD81, SLC40A1, CTSS, FCER1G, CXCL9
Myeloid 1	DC2 ($CD1C^+$)	1,394	2,940	13,398	S100B, HLA-DQB2, FCER1A, CD1A, PKIB, NDRG2
Myeloid 2	pDC	826	2,240	8,726	PTGDS, GZMB, IGJ, TCL1A, LILRA4, PLAC8, ITM2C, IRF7, PPP1R14B, TCF4, TSPAN13, CLIC3, SELL, PLD4, UGCG, CCDC50, C12orf75, BCL11A, MZB1, PTCRA, SPIB, LRRC26, SMPD3, MAP1A, ALOX5AP, SCT, PTPRS, IL3RA, APP, CYB561A3, SEC61B, SPCS1, CLEC4C, SLC15A4, HIGD1A, NUCB2, MPEG1, LTB, GAPT, IDH3A, LAMP5, C10orf118, TRAF4, OFD1
Myeloid 3	Monocyte	545	2,241	9,043	S100A8, TIMP1, S100A9, IL1B, FCN1, VCAN, IL8, S100A12, APOBEC3B, G0S2, CCL2, S100A4, CCL20, APOBEC3A, SOD2, IL1RN, FPR1, C15orf48, EREG
Myeloid 4	DC3 ($CCR7^+$)	362	2,482	11,249	BIRC3, CCL19, FSCN1, CCR7, CCL22, MARCKSL1, TXN, CCL17, LAMP3, CRIP1, NUB1, IDO1, KIF2A, SAMSN1, ID2, CD83, CST7, RAB9A, LSP1, TBC1D4, EBI3, ANXA6, BTG1, GRSF1, CSF2RA, LAD1, PNRC1, GPR157, BASP1, PLA2G16, DUSP4, DAPP1, RAMP1, AOC1, NCCRP1, PPA1, MAP3K13, STK4, UBD, IL32, LY75, FAM60A, ERICH1, RPS27L
Myeloid 5	DC1 ($CLEC9A^+$)	227	2,606	12,918	RGCC, CPVL, CLEC9A, CPNE3, C1orf54, DNASE1L3, SNX3, CCND1, IRF8, LGALS2, CLNK, CST3, NAAA, SERPINB1, PPT1, XCR1
T cell 0	$CD4^+$ Tregs-1	3,146	2,143	6,894	NA
T cell 1	$CD8^+$ T-1	2,589	2,003	6,206	GZMH, GZMA, CCL5
T cell 2	$CD4^+$ Tconv-1	2,358	1,806	5,993	NA
T cell 3	$CD4^+$ Tconv-2	2,212	1,606	6,329	NA

T cell 4	$CD8^+$ T-2	2,142	1,802	5,889	NA
T cell 5	Dysfunctional $CD8^+$ T	1,356	2,418	8,177	CCL4, CCL3, GZMB, NKG7, GZMK, IFNG, HIST1H4C, LAG3, HAVCR2
T cell 6	$CD4^+$ Tregs-2	1,218	2,562	9,170	TNFRSF4, TNFRSF18, TNFRSF9, IL2RA, PKM, HSP90AB1, BATF
T cell 7	$CD4^+$ Tconv-3	1,124	2,151	6,995	CXCL13
T cell 8	$CD8^+$ T-3	888	1,941	6,782	IFIT3, ISG15, IFIT1, IFIT2, RSAD2, MX1, MT2A
T cell 9	NK cell	708	2,031	6,380	GNLY, TYROBP, KLRC1, FCER1G, XCL1, XCL2, KLRD1, CTSW, IFITM3, HOPX, KLRF1, KLRC2, PRF1
B cell 0	B cell-1	6,017	1,619	6,208	NA
B cell 1	B cell-2	5,353	1,672	5,478	NA
B cell 2	Plasma cell	2,022	2,430	9,970	IGJ, XBP1, MZB1, SSR4, DERL3, FKBP11, HSP90B1, ITM2C, SEC11C, PRDX4, FKBP2, HERPUD1, JUN, SSR3, VIMP, SDF2L1, SELK, SPCS2, MANF, C19orf10, SPCS3, HSPA5, IFI6, PPIB, RRBP1, SEC61B, DNAJB9, PIM2, SPCS1, CD63, PDIA4, ERLEC1, NUCB2, TNFRSF17, SEC61G, LMAN1, JSRP1, TMEM258, TMEM59, RPN2, RABAC1, SDC1, LGALS1, KDELR2, CRELD2, IFI27, SUB1, PSAP, GSTP1, CD38, NPC2, TMED10, ARF4, NEAT1, KRTCAP2, TMED9, SELM, PDIA6, SLAMF7, HM13, P4HB, SRGN, TMED2, TXNDC15, CD27, KDELR1, TRAM1, HLA-C, CD59, TMBIM6, TIMP1
B cell 3	$FCRL4^+$ memory B cell	1,890	2,257	8,655	FCRL4, ISG15
B cell 4	B cell-3	897	2,263	9,560	FABP5, HSP90AB1, ENO1, LDHA, NME1
B cell 5	Germinal center B cell	595	2,564	9,985	MARCKSL1, RGS13, HMGN1, TCL1A, NEIL1, HMCES, ACTG1, LRMP, LPP, GAPDH, SYNE2, SERPINA9, DAAM1, BCL7A, HMGN2, SERF2, SUGCT, PTTG1, SNX29P2, EZR, RFTN1, HMGA1, UCP2
B cell 6	B cell-4	579	2,727	16,830	IGLL5, HIST1H4C, HMGB2, STMN1, MKI67, KIAA0101, TUBA1B, TUBB, H2AFZ, TOP2A, TK1, DUT, UBE2C, CENPF, NUSAP1, TYMS, RRM2
