# Supplementary material for: Single-cell transcriptomics reveals regulators underlying immune cell diversity and immune subtypes associated with prognosis in nasopharyngeal carcinoma
Source: Cell Res. 2020 Jul 20;30(11):1024–42. doi: 10.1038/s41422-020-0374-x (PMC7784929; doi:10.1038/s41422-020-0374-x)
Supplement: Supplementary file 17 — Supplementary information, Table S5 [file 41422_2020_374_MOESM17_ESM.pdf]

Table S5. Five malignant signatures derived from multiple related NMF metagenes

Epi-differ 1		Epi-differ 2		Epi-differ 3		Cell cycling		Cell secretion	
CXCL11	FBP1	CYP2A6	RP11-400N13.3	RGS17	STATH	DEPDC1	FAM64A	BEST2	GNG4
CSF2	IGFBP5	CEACAM7	SPRR2D	RP11-734K21.3	KIF26A	DLGAP5	KIF4A	CPA3	NLRP7
SAA1	UGT2B7	AP001610.9	RP11-350J20.12	DSG4	CTD-2547H18.1	HJURP	KIF2C	MYBPC1	RPS14
SFN	LAMB3	RP11-272B17.2	UGT2B4	GPRC5D	RP11-317L10.1	PBK	CENPF	GNB2L1	PRR15L
TRAF1	BHLHE41	IL36G	SPNS2	TGM3	ITLN1	TOP2A	AURKB	SHH	CAPN14
SOD2	KRT6A	HEPHL1	SERPINB2	AC006262.5	PHGR1	GTSE1	KIF14	RIMS2	RSPO4
MMP28	S100A8	PSAPL1	SOX8	RALYL	PLAC4	ASPM	NUF2	CTD-2521M24.8	ZBTB16
BDKRB1	CRYAB	C4orf19	IL36RN	HTR3B	IL37	CDC20	ESCO2	COL10A1	LMO3
KRT6B	RNF19B	SPRR2E	RP11-78C3.1	RP1-63G5.7	ELF5	CEP55	NUSAP1	EGFL6	LCE1E
ANGPTL4	CSTB	SPRR2A	SEMG1	TMPRSS11BNL	TRIML1	CKAP2L	NCAPG	DNAAF1	SRGN
CCL5	AKR1B10	GPR64	SPRR2B	C2orf72	TMEM45B	HMMR	CDCA3	SDPR	NHP2
LCE1C	SAA2	VNN1	TMEM255A	WI2-85898F10.1	RP11-120C12.3	NEK2	CDCA5	SMAD5-AS1	ACHE
CHI3L2	OASL	GPR110	RP11-211G23.2	FETUB	TMPRSS11B	BIRC5	CENPE	TUBB2B	PCDH11X
IFI44L	TREM2	RP11-798K3.3	APOBEC3A	SPINK1	SPRR3	CCNA2	KIF20A	PYDC1	RP4-639F20.1
MRGPRX3	KRT6C	IL19	RP11-462G2.1	SYTL5	C13orf45	MKI67	CENPA	RAMP2	INSM1
MRGPRX3	KRT6C	CYP2A6	RP11-400N13.3	RGS17	STATH	DEPDC1	FAM64A	BEST2	GNG4
