# Supplementary material for: Single-cell transcriptomics reveals regulators underlying immune cell diversity and immune subtypes associated with prognosis in nasopharyngeal carcinoma
Source: Cell Res. 2020 Jul 20;30(11):1024–42. doi: 10.1038/s41422-020-0374-x (PMC7784929; doi:10.1038/s41422-020-0374-x)
Supplement: Supplementary file 20 — Supplementary information, Table S8 [file 41422_2020_374_MOESM20_ESM.pdf]

Table S8. Clinical features of NPC Cohort B (n =128)

Variable	No. of patients
Age (years)	
Median (range)	42 (21-72)
Sex	
Male	94
Female	34
With smoking history	
Yes	44
No	84
Clinical stage	
I-II	9
III-IV	119
Plasma EBV DNA level	
I-II	9
III-IV	119
