# Supplementary material for: Single-cell transcriptomics reveals regulators underlying immune cell diversity and immune subtypes associated with prognosis in nasopharyngeal carcinoma
Source: Cell Res. 2020 Jul 20;30(11):1024–42. doi: 10.1038/s41422-020-0374-x (PMC7784929; doi:10.1038/s41422-020-0374-x)
Supplement: Supplementary file 22 — Supplementary information, Table S10 [file 41422_2020_374_MOESM22_ESM.pdf]

Table S10. Primers for real-time PCR

Gene		Sequence (5' to 3')
Human *RUNX1*	Forward	CTGCCCATCGCTTTCAAGGT
	Reverse	GCCGAGTAGTTTTCATCATTGCC
Human *BACH1*	Forward	TCTGAGTGAGAACTCGGTTTTTG
	Reverse	CGCTGGTCATTAAGGCTGAGTAA
Human *NR1H3*	Forward	CCTTCAGAACCCACAGAGATCC
	Reverse	ACGCTGCATAGCTCGTTCC
Human *TFEC*	Forward	TTAGAGCAGGCTAACAGGCGAC
	Reverse	ATCAACCGTGCCAAGTGAAGCC
Human *GAPDH*	Forward	CCTGAGCTGAACGGGAAGC
	Reverse	AGGTGGAGGAGTGGGTGTCG
